# Quality assurance and curricular development of medical faculties using graduate surveys: Challenges – proposal of a core questionnaire – implementation guide

**DOI:** 10.3205/zma001531

**Published:** 2022-02-15

**Authors:** Marianne Giesler, Johanna Huber, Volker Paulmann

**Affiliations:** 1 Freiburg, Germany; 2 LMU München, Klinikum der Universität München, Institut für Didaktik und Ausbildungsforschung in der Medizin, München, Germany; 3 Medizinische Hochschule Hannover, Studiendekanat - Bereich Evaluation & Kapazität, Hannover, Germany

**Keywords:** graduate surveys, quality assurance, curriculum development

## Abstract

Graduate surveys provide valuable information to further improve and develop an academic study program. The aim of this project report is to point out the relevance of these surveys for medical schools and to offer guidance on planning and implementing such surveys so that comparable evaluations of medical degree programs are possible.

The authors of this project report were asked by the MFT working subgroup on *Quality Assurance in Education* to generate quality recommendations for surveying medical graduates. To accomplish this, the questionnaires used by the medical schools to survey graduates were closely inspected and analyzed. A questionnaire containing core and optional questions was created as part of this project. A comprehensive matrix was also developed listing all of the procedural elements of graduate surveys.

## Zusammenfassung

Befragungen von Hochschulabsolventinnen und -absolventen liefern wertvolle Hinweise zur Verbesserung und Weiterentwicklung eines Studiengangs. Ziel des vorliegenden Projektberichts ist es, die Relevanz dieser Befragungen für medizinische Fakultäten aufzuzeigen und gleichzeitig Hilfestellung bei der Planung und Durchführung dieser Befragungen zu bieten, um so eine vergleichende Evaluierung von medizinischen Studiengängen zu ermöglichen.

Die Autorinnen und der Autor dieses Projektberichts wurden von der MFT-*Unterarbeitsgruppe* (UAG) *Qualitätssicherung Lehre* beauftragt, Qualitätsempfehlungen für die Befragung von Medizin-Absolventinnen und -Absolventen zu erarbeiten. Hierzu wurden die an medizinischen Fakultäten eingesetzten Fragebögen gesichtet und bewertet, mit denen Hochschulabsolventinnen und -absolventen befragt werden. Im Rahmen dieses Projekts entstand ein Fragebogen mit Kern- und optionalen Fragen. Zudem wurde eine Matrix entwickelt, die Verfahrenselemente von Befragungen von Absolventinnen und Absolventen enthält, beginnend mit „A wie Adressrecherche“ bis „Z wie Zeitraum der Befragung“.

## Introduction

Graduate surveys have been used more or less regularly by German-speaking universities since the 1970s [1]. The results of these surveys can provide information about the professional success and current whereabouts of program graduates, as well as give feedback on their experiences of the study program and the skills required in their professional life. These surveys are a source of valuable information to further develop an academic study program.

Since the end of the 1980s there has been an increase in the number of graduate surveys. On the one hand, this is a consequence of the recommendation given at that time by the West German conference of university rectors (Westdeutschen Rektorenkonferenz) which advised the universities to include graduate surveys alongside other course and teacher evaluations [2]; on the other, this increase was forced at the end of the 1990s with the general changes to universities that came with the Bologna Process and the associated compulsory quality assurance procedures [3]. Against this background, different projects have established themselves over the years, such as the studies on graduates since 1989, which have been done by the *Deutsches Zentrum für Wissenschaft und Forschung* (DZHW, formerly HIS) [https://www.dzhw.eu/forschung/projekt?pr_id=467]. Another is the *Kooperationsprojekt Absolventenstudien* (KOAB) initiated in 2007 by the *International Center of Higher Education and Research Kassel* (INCHER Kassel) and the *Netzwerk Absolventenforschung*. This collaborative project has been continued since 2016 by the *Institut für angewandte Statistik* (ISTAT) as independent spin-off from INCHER [https://istat.de/de/koab_a.html]. In addition to these nation-wide projects, there are associations at the state level, such as the *Sächsische Absolventenstudie* [4], the collaborative project *Baden-Württembergische Absolventenstudie* [5], and the *Bayerische Absolventenstudie* conducted by the *Bayerischen Staatsinstituts für Hochschulforschung und Hochschulplanung* (IHF) [https://www.bap.ihf.bayern.de/bas/aktuelles]. Since 2008 graduates have been regularly surveyed by the Freiburg Medical Faculty as part of the *Kompetenznetz Lehre in der Medizin Baden-Württemberg* [http://www.medizin-bw.de/]. Surveys regarding the university degree programs in medicine, veterinary medicine, and dental medicine have been conducted in Bavaria since 2015 as part of the *Bayerische Medizinabsolventenstudie* (MediBAS) [https://www.bap.ihf.bayern.de/medibas].

### Area of application for the surveys

The first graduate surveys were primarily conducted to receive differentiated information about the transitions between higher education and professional life, for instance, on employment situations and unemployment rates. As time progressed, however, there was increasing focus placed on questions asking about the quality of university study [1], [2], [6], [7]. A study done at Bavarian universities showed that 77% of the universities view graduate surveys as an important tool for quality assurance [8]. For 55% of the surveyed universities this type of survey is relevant for strategic university development. Forty-one percent use the results for the career center and/or university marketing, and 32% for academic advising.

Table 1 (Tab. 1) shows the diversity of survey use, the focus and purpose of which can differ depending on the university.

**Figure 1:**
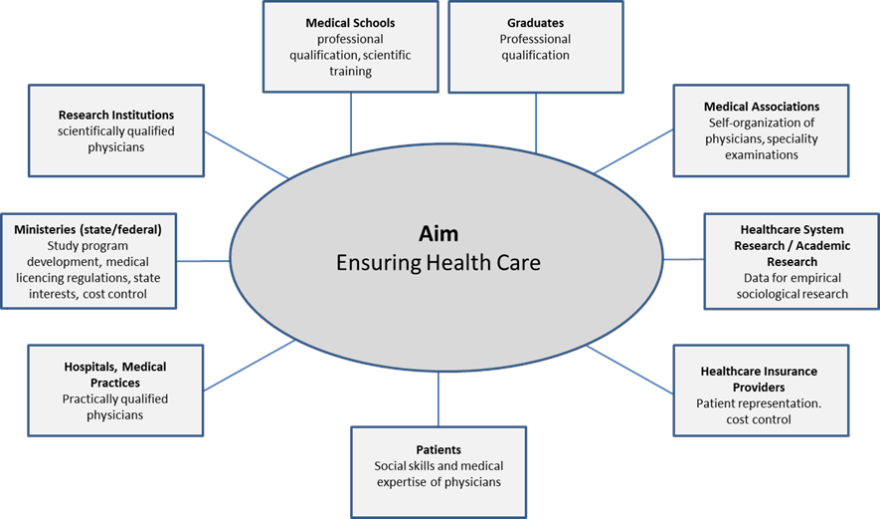
Stakeholders in medical education who use information from graduate surveys, or contribute to them, or gather the information themselves.

In addition to the internal interests of the universities connected with gathering such data comes the need for information on the part of external institutions. This reflects the complex constellation in which medical education and political, academic, and professional associations, as well as healthcare bodies, work together. This expressly involves ensuring healthcare with the best-trained physicians possible as an overall societal goal (see figure 1 (Fig. 1)). Graduate surveys are in part laid down in cooperative agreements between ministries and universities – as is the case in Nordrhein-Westfalen [https://www.mkw.nrw/sites/default/files/documents/2018-10/hochschulvereinbarung_nrw_2021_ohne_unterschrift.pdf]. Other interest groups are active in giving direct support, for example, as a partner in collaborative research (healthcare system research), as the instigator for independent research projects (statutory health insurance providers), or as the recipient of quality assurance measures in the area of healthcare (patients, clinics, hospitals).

The heterogeneity of the areas of application and purposes of the data collected through graduate surveys is reflected in the questionnaires that have been used to date. Each university distinguishes itself in a variety of ways based on regional factors, program-specific curricula, or historical background, all of which sensibly call for tailoring the questionnaires. On the other hand, the different questionnaires and survey time points make it difficult to compare medical schools with each other. When evaluating different teaching strategies – and the resulting academic outcomes – the ability to make such comparisons would be necessary. In regard to this, the Wissenschaftsrat (German Council of Science and Humanities) had already stated in 2014 that objectifiable evaluation criteria and evaluation methods for a comparable evaluation of medical education nationally and internationally have not yet been sufficiently established [9].

## Project description

A working subgroup based on the routinely held meetings of the medical school deans in Germany (*Medizinischer Fakultätentag*, or MFT) specifically addressed quality assurance in education by developing a list of criteria for assessing the quality of education. In the course of doing this work, it became clear that particularly when conducting graduate surveys no quality criteria existed. The authors of this report, who have gathered wide-ranging experience with graduate surveys in their university networks, were asked in 2018 to examine and assess the existing procedures and to use the results to make recommendations for planning and implementing graduate surveys. 

This report describes the working group’s results from February 2018 to October 2019 which are presented in the context of research on graduate surveys in the German-speaking countries.

## Method

The first step entailed inspecting several of the questionnaires that have been used by German medical schools. In part, these were variations of the physician surveys used by INCHER or ISTAT and different versions of the surveys used in Bavaria, Baden-Württemberg and Lower Saxony. The working group then checked the extent to which these questionnaires were the same in regard to the dimensions they contained (see table 2 (Tab. 2)). The dimensions were compiled as a list, and the questions frequently asked on these questionnaire versions were then assigned to the dimensions. In the next step each member of the working group was tasked with prioritizing questions that should be obligatory when surveying graduates. A question was classified as “obligatory” if the following conditions were met:

- It had a direct bearing on quality assurance processes or appeared relevant for a comparison concerning the quality of input, processes, and outcomes of the education. In general, these were questions with which information is collected that is regularly asked for in connection with assessments, both internal (by university committees) and external (accreditations, ministries, *Wissenschaftsrat*, etc.). Or it is discussed within education research as being central criterion for education quality [10].
- All three members of the working group unanimously found it to be essential. If only two members agreed, the relevance of the question was discussed critically and then a decision was made about whether it could be classified as “obligatory” or “not obligatory.” If questions were formulated in a similar way, one version was selected through a consensus procedure.

The questions selected in this manner were designated as “core questions”. Classified as “optional” were questions that more deeply probe relevant aspects regarding education quality or professional development, but that are more regionally limited in their informative value and therefore do not need to be essential for all surveys. Overall, an effort was made to basically reduce the number of questions since the questionnaires that were reviewed were usually very extensive.

Parallel to examining the questionnaires, best-practice examples were collected from the group’s own questionnaires to illustrate effective resource use, high response rates, and practicability (data protection issues etc.) The examples were sorted by topic and supplemented with recommendations from the literature. Based on this, a matrix of procedural elements for graduate surveys was created that includes concrete instructions for conducting these surveys, from finding current addresses to defining the time period for the survey.

The work process included the participation of experts from the project *Karriereverläufe* von *Ärztinnen und Ärzten* *in der fachärztlichen Weiterbildung* (KarMed: funded by the *Bundesministerium für Bildung und Forschung* (Federal Ministry of Education and Research, or BMBF)) and the *Kassenärztliche Bundesvereinigung* (Association of Statutory Health Physicians, or *KBV*) [11]. This allowed for discussions of the intermediate results. The tentative conclusion of the project activities is the presentation and discussion of the results at the 12^th^ meeting of the university deans of studies in November 2019 in Berlin.

## Results

### Questionnaires with core questions

The comparison of the survey instruments used by the medical schools revealed the following aspects: In regard to the structure of the questionnaires and their central elements, it was seen that these almost equally cover a retrospective view of university study and the professional experience gathered since then. However, the range covered by the questions within these two training phases varies substantially. Furthermore, there is a wide spectrum of specific questions and different formulations that make comparisons of the answers difficult.

Based on the work steps described above (see Method), a questionnaire was created with core questions (obligatory questions) for medical graduates (medicine and dental medicine). These core questions were meant to form the basis of each graduate survey in order to enable meaningful comparisons. Each medical school would have the opportunity to add optional questions reflecting the university’s specific interests. Table 2 (Tab. 2) provides an overview of the topics covered by the core questions and includes optional questions. The questionnaire can be downloaded as an attachment 1 (german version) to this report.

#### Matrix of procedural elements for graduate surveys

The matrix on conducting graduate surveys covers 20 topics. In the first section on researching addresses, it explains how survey participants’ addresses can be updated; the last section describes the concepts of “time point” and “time period” and when a graduate survey should be conducted. Following this are practical recommendations and options for each topic. Finally, potential problems are identified. Supplemental references to the literature are given at the end of the sections on each topic.

This matrix is not exhaustive, but is rather to be viewed as a preliminary list open to the addition of other evidence-based recommendations for conducting graduate surveys. This matrix is designed to provide guidance for medical schools which have previously had little or no experience with carrying out such surveys.

The matrix can be downloaded as an attachment 2 (german version) to this report.

## Discussion and outlook

In the fall of 2018 the authors of this report were asked by an MFT working subgroup to generate recommendations for planning and conducting graduate surveys. Two products were developed in the course of this project and are the subject of this report. To enable a comparative evaluation of medical education in Germany as requested by the *Wissenschaftsrat*, a questionnaire was developed for use by medical schools. The core questions on this questionnaire are meant to ensure that all of the information relevant to quality assurance is collected. Optional questions that are of interest to specific universities can be added. A matrix based on best-practice examples collected by the authors of this report and supplemented with examples from the literature was created to serve as an additional aid in planning and conducting the surveys. These two products should not only contribute to the further development of medical education and its quality assurance, but also allow for comparisons between medical schools regarding the educational situation. The latter should be advanced for the following reasons:

1. The use of programs to qualify medical teachers in the German-speaking countries [12], [13] and the newly established professorships for medical education at the universities create the conditions under which the number of research projects to improve education can be increased. As a consequence, more questions about basic aspects of curriculum development can be investigated scientifically, as can the inputs, the teaching and learning processes, and the outcomes of education. Such questions gain more significance if they are addressed through multicenter approaches. Graduate surveys that enable cross-university comparisons can be helpful in this regard.
2. The growing importance of medical education and the research on it are also reflected in the new version of the German medical licensing regulations (*Ärztlichen Approbationsordnung,* or ÄApprO). The number of sections in the draft legislation (an increase from 44 to 183 sections) shows that the complexity of education-related issues has increased substantially. The National Competency-based Catalogue of Learning Objectives for Undergraduate Medical Education (Nationale* Kompetenzbasierte Lernzielkatalog*, or NKLM), which has undergone a development process of many years, has now been integrated into the ÄApprO. Given this focus on competencies, the question about the measurability of competency arises. In addition to the student outcomes on the second part of the M2 state medical exam, more data will be needed to verify that educational objectives have been achieved – regardless of whether they represent practical or scientific competencies in medicine. Self-assessments by graduates of their own competencies [14] could, alongside scores on practical assessments (OSCE), which, by the way, should also be expanded, provide information regarding the quality of education.
3. Interregional research projects that draw on the results of graduate surveys already exist. For instance, within the scope of the project *Studierendenauswahl Verbund (stav)* [15], cross-university postgraduate data is being used to draw conclusions about the success of the admissions process and the different groups of admitted students. The requirements for robust data on courses of study and professional careers will also increase as a result of the statutory stipulations for structuring the admission processes.

In addition to the ongoing use and potential uses for graduate surveys, there are, however, some obstacles and limits which are briefly described here:

1. When planning graduate surveys, the question always arises as to how to balance successfully between questions that relate to the current moment (e.g., work/life balance of young physicians) and questions that are of interest over a longer period of time (e.g., developments regarding doctoral degrees in medicine). A questionnaire containing the same questions over many years of student cohorts will be necessary to make reliable internal and external comparisons. On the other hand, new questions will need to be integrated in order to remain current. For instance, in the future there will be questions about the effects of the coronavirus pandemic on medical education that will of necessity complement older questions. So that a questionnaire is answered in full by respondents, its length should be carefully calculated. Analysis of the stopping points on incomplete questionnaires for earlier surveys (dropout analysis), for instance, can provide valuable information about possible optimization [16]. The use of core questions that remain an unchanged part of all surveys over a longer period of time and the potential inclusion of optional questions can be helpful.
2. When calling for a benchmark system, it is often underestimated how much results and processes are influenced by factors that make comparisons difficult. For a wide variety of reasons it is virtually impossible to measure the actual teaching at medical schools as a determining factor. In addition, there are many factors external to the university which also help to steer educational success. Van den Bussche et al. [17] were able to show that state exam scores can be influenced by factors such as the size of the university, staffing at the university, an examinee’s academic record in secondary school, and their citizenship. A uniform collection of data at the medical schools is important to take these and other potential influencing factors into account.
3. Because the university rectors are usually in charge of graduate surveys, they also have a decisive effect on the form and content of the questionnaires. It is often the case when developing a uniform, interdisciplinary questionnaire that special aspects of individual subjects are not taken into consideration. The need for university leaders to compare the results of the different subjects with each other is understandable, but it does not do justice to the needs and interests of the medical schools because the structures and conditions of a degree program in medicine, dentistry, or veterinary medicine differ very strongly from the degree programs in other fields of study. A comparison of the results with other medical schools is limited when a uniform, university-specific questionnaire is used for all subjects. It must also be assumed that the acceptance of such surveys will be negatively affected, not only in terms of those surveyed, but also those who receive the reports. It would be possible to better account for the interests of the medical schools by having medical faculty participate on the relevant steering committees. Agreements could also be reached that are structured so that medical schools carry out surveys in coordination with rectors who then, in turn, make the survey data available. Several medical schools have indicated that this is their approach, according to the results of a survey conducted at a German-speaking medical school [7].
4. The issue of a survey’s representativity is crucial for the acceptance of the results. The tracking of foreign careers (including foreign students who do not work in Germany after completing their studies) is limited due to the difficulties in finding and then contacting these graduates. This group is generally underrepresented in surveys. Moreover, with response rates of 30-50% it cannot be ruled out that the composition of the samples does not correspond with some relevant characteristics of the target population [18]. It may be assumed that - among other factors - the increasing use of surveys in the evaluation of university teaching contributes to a decline in response rates and compromises data quality [19], [20].
5. From a methodological viewpoint, carrying out panel surveys, with which the professional development of graduates is followed over a long period of time, is worth pursuing. In recent years, only the KarMed studies [11], as a prospective study on career paths in medicine, alongside the data collected by the DZHW, have done justice to these needs. Anchoring resources is desirable to ensure ongoing research on graduates at the medical schools.

## Take-home message

In summary, suitable instruments are available in the form of the core questionnaire and the matrix outlining all of the procedural elements to assist medical schools in conducting graduate surveys. Both instruments can be downloaded as attachment 1 (german version) and attachment 2 (german version) to this report.

## Competing interests

The authors declare that they have no competing interests. 

## Supplementary Material

in German: Befragung von Absolventinnen und Absolventen des Studiengangs Humanmedizin

Procedural elements of graduate surveys

## Figures and Tables

**Table 1:**
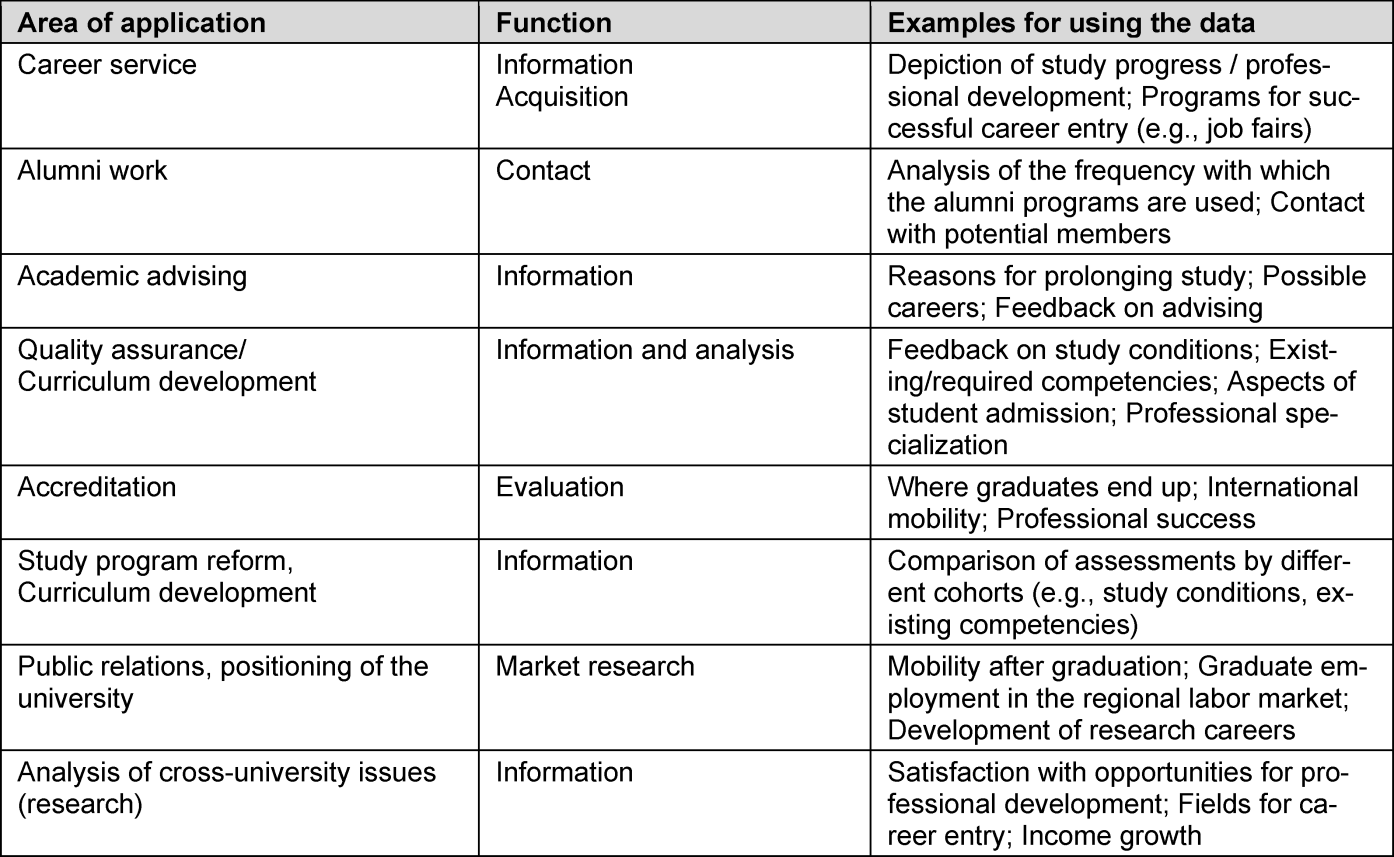
Areas of application and the function of graduate surveys at universities based on Janson [21]

**Table 2:**
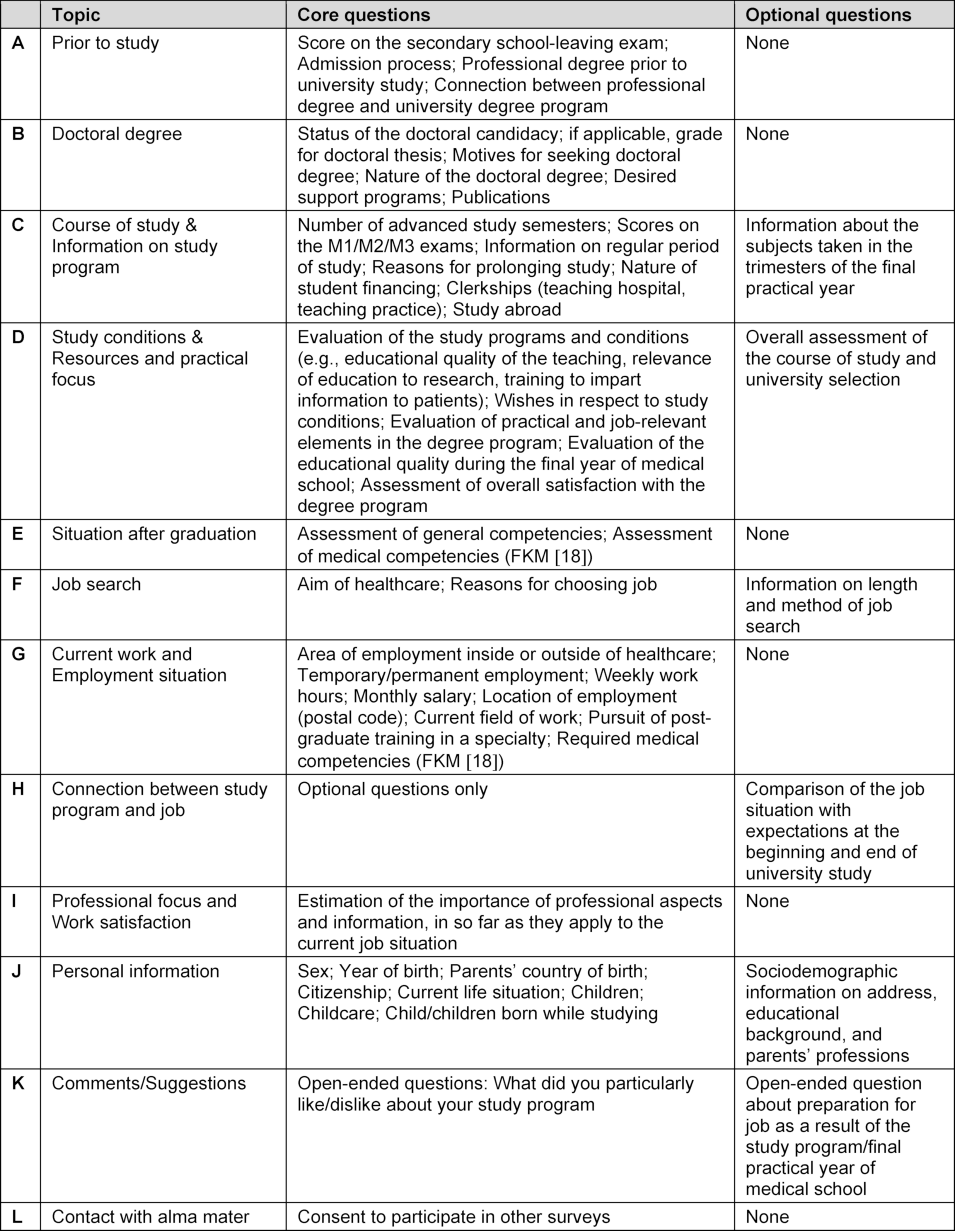
Core and optional questions for surveying graduates (see attachment 1 in German) according to topic.
