# Supplementary material for: Quality assurance and curricular development of medical faculties using graduate surveys: Challenges – proposal of a core questionnaire – implementation guide
Source: GMS J Med Educ. 2022 Feb 15;39(1):Doc10. doi: 10.3205/zma001531 (PMC8953197; doi:10.3205/zma001531)
Supplement: in German: Befragung von Absolventinnen und Absolventen des Studiengangs Humanmedizin [file JME-39-10-s-001.pdf]

Befragung von Absolventinnen und Absolventen des Studiengangs Humanmedizin

Befragung der Absolventinnen und Absolventen des
Wintersemesters 20XX / 20XX und des Sommersemesters 20XX

Kontakt:

Universität/Medizinische Fakultät xxxx

XXXXXXX
XXXXXXXXXXXXXXXXXX
XXXXX@XXXXXXXX.de
Tel.: +49 XXXXXXX

Dieser Fragebogen enthält sogenannte Kernfragen (im Text mit KERN gekennzeichnet), die für alle Fakultäten verpflichtend sein sollen und sogenannte optionale Fragen (im Text mit OPTIONAL gekennzeichnet).

Anhang 1 zu Giesler M, Huber J, Paulmann V. Quality assurance and curricular development of medical faculties using graduate surveys: Challanges - proposal of care questionnaire - implemention guide. GMS J Med Educ. 2022;39(1):Doc10. DOI: 10.3205/zma001531

Hinweise zum Ausfüllen des Fragebogens

Bitte beantworten Sie die Fragen in der vorgegebenen Reihenfolge und kreuzen Sie die entsprechende Antwort an bzw. füllen Sie die dafür vorgesehenen Textfelder möglichst leserlich aus.

Verwenden Sie hierzu bitte nur Kugelschreiber oder Füllfederhalter, keinen Bleistift oder hellen Faserstift.

In den meisten Fällen ist ein Kästchen anzukreuzen. Falls mehrere Kästchen angekreuzt werden können, ersehen Sie dies aus dem Hinweis "Mehrfachnennungen möglich".

Falls Sie versehentlich das falsche Kästchen angekreuzt haben: schwärzen Sie bitte die falsche Markierung und markieren Sie das richtige Kästchen.

Gelegentlich werden Sie im Fragebogen aufgefordert, für Sie nicht zutreffende Fragen zu überspringen; dies geschieht mit dem Hinweis: "→ Bitte weiter mit Frage ...".

Falls der Platz für Ihre Angaben bei einzelnen Fragen nicht ausreicht, machen Sie diese bitte auf einem gesonderten Blatt und fügen Sie dieses dem Fragebogen bei.

Nachfolgend finden Sie einen Überblick über den Inhalt des Fragebogens:

A Vor dem Studium

B Promotion

C Studienverlauf & Angaben zum Studium

D Studienbedingungen & Ausstattung und Praxisorientierung

E Die Situation nach Studienabschluss

F Beschäftigungssuche

G Derzeitige Tätigkeit und Beschäftigungssituation

H Zum Zusammenhang von Studium und Beruf

I Berufliche Orientierungen und Arbeitszufriedenheit

J Angaben zur Person

K Kommentare / Anregungen

L Kontakte zu Ihrer Universität

Kommentare zum Fragebogen sind uns willkommen!

Anhang 1 zu Giesler M, Huber J, Paulmann V. Quality assurance and curricular development of medical faculties using graduate surveys: Challanges - proposal of care questionnaire - implemention guide. GMS J Med Educ. 2022;39(1):Doc10. DOI: 10.3205/zma001531

Hinweise zum Datenschutz

Die Daten werden von der Universität/der Medizinischen Fakultät XXXXXX erhoben. Ihre Teilnahme an der Befragung ist freiwillig und es entstehen Ihnen durch eine Nichtteilnahme keine Nachteile.

Es kann nicht ausgeschlossen werden, dass in bestimmten Fällen aus der Kombination Ihrer Angaben ein Rückschluss auf Ihre Person möglich ist. Dies liegt jedoch nicht in unserem Interesse.

Sämtliche von Ihnen im Fragebogen gemachten personenbezogenen Angaben werden vertraulich behandelt. Ihre personenbezogenen Daten werden nur zu Zwecken der Qualitätsentwicklung verwendet. Die Daten werden statistisch ausgewertet und die Ergebnisse in aggregierter Form in wissenschaftlichen Publikationen präsentiert. Aus den veröffentlichten Ergebnissen der Datenauswertung wird kein Rückschluss auf einzelne Teilnehmer der Befragung möglich sein.

Sie haben im Anschluss an die Befragung die Möglichkeit, freiwillige Angaben dazu zu machen, ob Sie an einer weiteren Befragung teilnehmen oder einen Ergebnisbericht einsehen möchten. Ihre für diesen Zweck angegebenen Kontaktdaten werden sofort nach Eingang des Fragebogens von diesem getrennt und an einem von Ihren sonstigen Antwortdaten getrennten Speicherort verwahrt und können nicht mit diesen in Verbindung gebracht werden. Zugang zum vollständigen Datensatz haben ausschließlich Mitglieder des Evaluationsteams der Medizinischen Fakultät in XXXX.

ENTWURF

Anhang 1 zu Giesler M, Huber J, Paulmann V. Quality assurance and curricular development of medical faculties using graduate surveys: Challanges - proposal of care questionnaire - implemention guide. GMS J Med Educ. 2022;39(1):Doc10. DOI: 10.3205/zma001531

A VOR DEM STUDIUM

1 Welche Durchschnittsnote hatten Sie in dem Zeugnis, mit dem Sie Ihre Studienberechtigung erworben haben?

Durchschnittsnote ______ (bitte Punktzahl ggf. in Note umrechnen; bei ausländischer Studienberechtigung die anerkannte Note) KERN

2 Über welches Verfahren haben Sie Ihre Zulassung zum Studium erhalten (in welcher „Quote" haben Sie sich beworben)? KERN

- ☐ Abiturnote(Abitur-Besten-Quote)
- ☐ Auswahlgespräch (oder ein anderes Auswahlverfahren der Hochschulen – AdH)
- ☐ Wartezeit
- ☐ Studienplätze für ausländische Bewerberinnen
- ☐ Sonstiges (Zweitstudienbewerber, Sanitätsoffizier der BW, Härtefälle)

3 Haben Sie vor der ersten Einschreibung einen beruflichen Abschluss erworben? KERN

- ☐ Ja, welchen (bitte angeben):
- ☐ Nein

4 Inwieweit stand dieser berufliche Abschluss in einem fachlichen Zusammenhang mit Ihrem Studium? KERN

- ☐ 1 – In sehr hohem Maße
- ☐ 2
- ☐ 3
- ☐ 4
- ☐ 5 – Gar nicht

B PROMOTION

5 Haben Sie eine Promotion begonnen oder abgeschlossen? KERN

- ☐ Ja, begonnen
- ☐ Ja, abgeschlossen
- ☐ Ja, aber inzwischen abgebrochen
- ☐ Nein (Filterfrage)

6 Mit welcher Note haben Sie Ihre Promotion abgeschlossen? KERN

- ☐ Summa cum laude
- ☐ Magna cum laude
- ☐ Cum laude
- ☐ Rite
- ☐ Sonstige *(bitte eintragen)*:……………………………………………………………………………………………

Anhang 1 zu Giesler M, Huber J, Paulmann V. Quality assurance and curricular development of medical faculties using graduate surveys: Challanges - proposal of care questionnaire - implemention guide. GMS J Med Educ. 2022;39(1):Doc10. DOI: 10.3205/zma001531

7 Warum haben Sie sich für eine Promotion entschieden? KERN

- ☐ da die Promotion in meinem Fach weitgehend üblich ist
- ☐ da ich ohne Doktortitel Nachteile auf dem Arbeitsmarkt befürchtete
- ☐ um später allgemein in der Forschung arbeiten zu können
- ☐ um fachlich dazuzulernen
- ☐ um während der Promotion forschen zu können
- ☐ um mich intensiver mit dem speziellen Thema meiner Promotion beschäftigen zu können
- ☐ um mir eine wissenschaftliche Karriere offen zu halten
- ☐ um evidenzbasierte Medizin besser praktizieren und nachvollziehen zu können
- ☐ um ein höheres Einkommen zu erzielen als ohne Doktortitel
- ☐ für ein höheres gesellschaftliches Ansehen
- ☐ um von Patienten als kompetenter Arzt wahrgenommen zu werden
- ☐ um ein höheres Ansehen bei Kollegen zu haben

8 Welchen Charakter hat/hatte Ihre Promotionsarbeit? KERN

- ☐ Experimentell (Auswertung eigener experimentell gewonnener Daten)
- ☐ Klinisch mit direkter Patientenbeteiligung
- ☐ Klinisch ohne direkte Patientenbeteiligung (statistische Auswertung nicht selbst erhobener Daten)
- ☐ Nicht-klinisch empirisch (mit Datenerhebung, z.B. Fragebogen)
- ☐ Nicht-klinische Literaturarbeit
- ☐ Sonstiges *(bitte eintragen)*: ....

ENTWURF

9 In welchen Phasen Ihrer Promotion hätten Sie sich Unterstützung gewünscht bzw. wünschen Sie sich Unterstützung? KERN

- ☐ Ausarbeitung der Fragestellung
- ☐ Strukturierung des Forschungsprozesses
- ☐ Datenerhebung
- ☐ Methodik/Statistik
- ☐ Abfassen der Arbeit (Formulierung, Einbinden von Literatur)
- ☐ Sonstiges:

10 Wie beurteilen Sie die Betreuung während Ihrer Promotion? KERN

11 Haben Sie die Ergebnisse Ihrer Dissertation in mindestens einer wissenschaftlichen Zeitschrift veröffentlicht? KERN

☐ Ja, als →

- ☐ Erstautor: in welcher Zeitschrift/welchen Zeitschriften? (bitte angeben)
- ☐ Koautor: in welcher Zeitschrift/welchen Zeitschriften? (bitte angeben)

Impact-Punkte:

☐ Nein

Anhang 1 zu Giesler M, Huber J, Paulmann V. Quality assurance and curricular development of medical faculties using graduate surveys: Challanges - proposal of care questionnaire - implemention guide. GMS J Med Educ. 2022;39(1):Doc10. DOI: 10.3205/zma001531

C STUDIENVERLAUF & ANGABEN ZUM STUDIUM

12 Wie viele Fachsemester haben Sie insgesamt in dem Studiengang studiert, den Sie im Wintersemester 2016/17 oder Sommersemester 2017 abgeschlossen haben? (ohne Urlaubssemester; einschließlich Semester im selben Fach an einer anderen Hochschule im In- und Ausland) Kern

Anzahl der Fachsemester:

13 Welche Noten haben Sie im Studium erhalten? Kern

13a HumanmedizinerInnen:

☐,☐	☐,☐	☐,☐	☐,☐
M1 (Physikum) schriftlich	M1 (Physikum) mündlich-praktisch	M2 Schriftlich	M2 bzw. M3 mündlich-praktisch

☐ Ich habe keine M1-Prüfung abgelegt, da ich an einem Modellstudiengang teilgenommen habe

13b ZahnmedizinerInnen:

☐,☐	☐,☐	☐,☐
Naturwissenschaftliche Prüfung	Zwischenprüfung Zahnmedizin	Abschlussnote Zahnärztliche Prüfung

14 Haben Sie Ihr Medizinstudium in der Regelstudienzeit abgeschlossen? Kern

☐ Ja → *Bitte weiter mit Frage 29*

☐ Nein: Es hat länger als die Regelstudienzeit gedauert

15 Warum haben Sie länger studiert als in der Regelstudienzeit vorgesehen? *(Mehrfachnennungen möglich)* Kern

☐ **Organisatorische Rahmenbedingungen des Studiums** Nichtzulassung zu Lehrveranstaltungen (z.B. wegen fehlender räumlicher oder personeller Kapazitäten) Schlechte Koordination der Studienangebote (Überschneidung von Lehrveranstaltungen etc.)

☐ Nicht bestandene Prüfungen

☐ Änderung / Umstellung der Prüfungs-, Studienordnung bzw. -struktur

☐ Nichtzulassung zu einer / mehreren Prüfungen (z.B. wegen fehlender personeller Kapazitäten oder Überschneidung von Prüfungsterminen)

☐ Hohe Anforderungen im Studiengang

☐ Hochschulwechsel

☐ Promotion

☐ Auslandsaufenthalt(e)

☐ Erwerbstätigkeit(en)

☐ Zusätzliches Studienengagement (habe mich um über den Studiengang hinausgehende Qualifikationen bemüht, Zweitstudium) Breites fachliches (inhaltliches, wissenschaftliches) Interesse (habe Veranstaltungen außerhalb meines Studienganges besucht)

☐ Engagement in Selbstverwaltungsgremien meiner Hochschule

☐ Gesellschaftspolitisches Engagement außerhalb des Studiums

☐ Persönliche Gründe (z.B. fehlendes Studieninteresse, Motivation empfundene hohe Anforderungen etc.)

☐ Familiäre Gründe (z.B. Schwangerschaft, Kinder, Pflege von Angehörigen etc.)

☐ Sonstiges *(bitte angeben)*: ………

Anhang 1 zu Giesler M, Huber J, Paulmann V. Quality assurance and curricular development of medical faculties using graduate surveys: Challanges - proposal of care questionnaire - implemention guide. GMS J Med Educ. 2022;39(1):Doc10. DOI: 10.3205/zma001531

16 Wie haben Sie Ihr Studium finanziert? Bitte verteilen Sie 100% auf die folgenden Finanzierungsquellen Kern

- ☐ Unterstützung durch Eltern oder andere Verwandte
- ☐ Unterstützung durch den (Ehe-)Partner/die (Ehe-)Partnerin
- ☐ Ausbildungsförderung nach dem BaFöG
- ☐ Eigener Verdienst aus Tätigkeiten während der Vorlesungszeit und/oder der vorlesungsfreien Zeit
- ☐ Kredit (z.B. Bildungskredit von der KfW Bankengruppe; Kredit zur Studienfinanzierung von einer Bank/Sparkasse oder von Privatpersonen)
- ☐ Stipendium
- ☐ Eigene Mittel, die vor dem Studium erworben/angespart wurden
- ☐ Andere Finanzierungsquelle:

17 In welcher Institution haben Sie Ihr praktisches Jahr vorwiegend absolviert? Kern

- ☐ Trifft nicht zu: Ich habe kein Praktisches Jahr absolviert
- ☐ Universitätsklinikum
- ☐ Akademisches Lehrkrankenhaus
- ☐ Akademische Lehrpraxis
- ☐ Sonstiges:

18 HumanmedizinerInnen: Haben Sie Teile Ihres PJs in einem akademischen Lehrkrankenhaus (nicht Universitätsklinikum) absolviert? Kern

- ☐ Ja (*bitte Fachrichtung auswählen, Mehrfachantworten möglich*) OPTIONAL
  - ☐ Allgemeinmedizin
  - ☐ Anästhesiologie
  - ☐ Arbeitsmedizin / Sozialmedizin
  - ☐ Augenheilkunde
  - ☐ Chirurgie
  - ☐ Dermatologie / Venerologie
  - ☐ Gynäkologie und Geburtshilfe
  - ☐ Hals-, Nasen- und Ohrenheilkunde
  - ☐ Humangenetik
  - ☐ Infektiologie / Immunologie
  - ☐ Innere Medizin
  - ☐ Kinder- und Jugendpsychiatrie
  - ☐ Kinderheilkunde / Kinder- und Jugendmedizin
  - ☐ Klinische Pharmakologie / Pharmakotherapie
  - ☐ Mikrobiologie
  - ☐ Neurochirurgie
  - ☐ Neurologie
  - ☐ Notfallmedizin
  - ☐ Nuklearmedizin / Strahlenbehandlung / Strahlenschutz
  - ☐ Öffentliches Gesundheitswesen
  - ☐ Orthopädie / Unfallchirurgie
  - ☐ Palliativmedizin
  - ☐ Pathologie

Anhang 1 zu Giesler M, Huber J, Paulmann V. Quality assurance and curricular development of medical faculties using graduate surveys: Challanges - proposal of care questionnaire - implemention guide. GMS J Med Educ. 2022;39(1):Doc10. DOI: 10.3205/zma001531

- [ ] Pharmakologie / Toxikologie
- [ ] Psychiatrie und Psychotherapie
- [ ] Psychosomatik und Psychotherapeutische Medizin
- [ ] Radiologie
- [ ] Rechtsmedizin
- [ ] Sportmedizin
- [ ] Tropenmedizin
- [ ] Urologie
- [ ] Virologie
- [ ] Sonstiges und zwar: ....

- [ ] Nein

19 HumanmedizinerInnen: Haben Sie Teile Ihres PJs in einem Universitätsklinikum absolviert? Kern

- [ ] Ja (*bitte Fachrichtung auswählen, Mehrfachantworten möglich*) OPTIONAL
  - [ ] Allgemeinmedizin
  - [ ] Anästhesiologie
  - [ ] Arbeitsmedizin / Sozialmedizin
  - [ ] Augenheilkunde
  - [ ] Chirurgie
  - [ ] Dermatologie / Venerologie
  - [ ] Gynäkologie und Geburtshilfe
  - [ ] Hals-, Nasen- und Ohrenheilkunde
  - [ ] Humangenetik
  - [ ] Infektiologie / Immunologie
  - [ ] Innere Medizin
  - [ ] Kinder- und Jugendpsychiatrie
  - [ ] Kinderheilkunde / Kinder- und Jugendmedizin
  - [ ] Klinische Pharmakologie / Pharmakotherapie
  - [ ] Mikrobiologie
  - [ ] Neurochirurgie
  - [ ] Neurologie
  - [ ] Notfallmedizin
  - [ ] Nuklearmedizin / Strahlenbehandlung / Strahlenschutz
  - [ ] Öffentliches Gesundheitswesen
  - [ ] Orthopädie / Unfallchirurgie
  - [ ] Palliativmedizin
  - [ ] Pathologie
  - [ ] Pharmakologie / Toxikologie
  - [ ] Psychiatrie und Psychotherapie
  - [ ] Psychosomatik und Psychotherapeutische Medizin
  - [ ] Radiologie
  - [ ] Rechtsmedizin
  - [ ] Sportmedizin

- ☐ Tropenmedizin
- ☐ Urologie
- ☐ Virologie
- ☐ Sonstiges und zwar: ....

☐ Nein

20 HumanmedizinerInnen: Haben Sie Teile Ihres PJs in einer akademischen Lehrpraxis absolviert? Kern

☐ Ja (*bitte Fachrichtung auswählen, Mehrfachantworten möglich*) OPTIONAL

- ☐ Allgemeinmedizin
- ☐ Anästhesiologie
- ☐ Arbeitsmedizin / Sozialmedizin
- ☐ Augenheilkunde
- ☐ Chirurgie
- ☐ Dermatologie / Venerologie
- ☐ Gynäkologie und Geburtshilfe
- ☐ Hals-, Nasen- und Ohrenheilkunde
- ☐ Humangenetik
- ☐ Infektiologie / Immunologie
- ☐ Innere Medizin
- ☐ Kinder- und Jugendpsychiatrie
- ☐ Kinderheilkunde / Kinder- und Jugendmedizin
- ☐ Klinische Pharmakologie / Pharmakotherapie
- ☐ Mikrobiologie
- ☐ Neurochirurgie
- ☐ Neurologie
- ☐ Notfallmedizin
- ☐ Nuklearmedizin / Strahlenbehandlung / Strahlenschutz
- ☐ Öffentliches Gesundheitswesen
- ☐ Orthopädie / Unfallchirurgie
- ☐ Palliativmedizin
- ☐ Pathologie
- ☐ Pharmakologie / Toxikologie
- ☐ Psychiatrie und Psychotherapie
- ☐ Psychosomatik und Psychotherapeutische Medizin
- ☐ Radiologie
- ☐ Rechtsmedizin
- ☐ Sportmedizin
- ☐ Tropenmedizin
- ☐ Urologie
- ☐ Virologie
- ☐ Sonstiges und zwar: ....

☐ Nein

Anhang 1 zu Giesler M, Huber J, Paulmann V. Quality assurance and curricular development of medical faculties using graduate surveys: Challanges - proposal of care questionnaire - implemention guide. GMS J Med Educ. 2022;39(1):Doc10. DOI: 10.3205/zma001531

21 Haben Sie während Ihres Studiums eine Zeit im Ausland/außerhalb Deutschlands verbracht? Kern

- ☐ Ja
- ☐ Nein

Welchen Zweck hatte Ihr Auslandsaufenthalt?

- ☐ Auslandssemester ja/nein
- ☐ Famulatur
- ☐ Praktisches Jahr
- ☐ Freiwilliges Praktikum
- ☐ Sonstiges

D STUDIENBEDINGUNGEN & AUSSTATTUNG UND PRAXISORIENTIERUNG

22 Wie beurteilen Sie die folgenden Studienangebote und -bedingungen in Ihrem Fach? Kern

Sehr gut 1	2	3	4	Sehr schlecht 5	
☐	☐	☐	☐	☐	Zeitliche Koordination der Lehrveranstaltungen
☐	☐	☐	☐	☐	Zugang zu erforderlichen Lehrveranstaltungen (z.B. Seminare, Übungen)
☐	☐	☐	☐	☐	Möglichkeit, die Studienanforderungen in der dafür vorgesehenen Zeit zu erfüllen
☐	☐	☐	☐	☐	System und Organisation von Prüfungen
☐	☐	☐	☐	☐	Aufbau und Struktur des Studiums
☐	☐	☐	☐	☐	Angebote zum Erwerb wissenschaftlicher Arbeitsweisen
☐	☐	☐	☐	☐	Training von mündlicher Präsentation
☐	☐	☐	☐	☐	Angebote zum Verfassen von wissenschaftlichen Texten
☐	☐	☐	☐	☐	Aktualität der vermittelten Methoden
☐	☐	☐	☐	☐	Didaktische Qualität der Lehre
☐	☐	☐	☐	☐	Fachliche Qualität der Lehre
☐	☐	☐	☐	☐	Fachliche Vertiefungsmöglichkeiten (Wahlfach)
☐	☐	☐	☐	☐	Forschungsbezug von Lehre
☐	☐	☐	☐	☐	Kontakte zu Lehrenden
☐	☐	☐	☐	☐	Kontakte zu Mitstudierenden
☐	☐	☐	☐	☐	Training zur Weitergabe von Informationen / Fachwissen an Patienten
☐	☐	☐	☐	☐	Training zur Weitergabe von Informationen / Fachwissen an Mitglieder anderer medizinischer Berufsgruppen *(z.B. Pflegekräfte, ...) einfügen*
☐	☐	☐	☐	☐	Angebote zum Erwerb kommunikativer Fähigkeiten
☐	☐	☐	☐	☐	Angebote zum Erwerb von Selbstmanagement-Fertigkeiten wie z. B. Zielsetzung, Selbstinstruktion, Selbstverstärkung
☐	☐	☐	☐	☐	Angebote zum Umgang mit ethischen Fragen in der Berufspraxis
☐	☐	☐	☐	☐	Einsatz moderner Lehrformen (z.B. POL, Skills-Lab, Rollenspiele mit Simulationspatienten)
☐	☐	☐	☐	☐	Breite des Lehrangebotes

23 In welchem Maße sollten die folgenden Angebote und Bedingungen Bestandteile des Studiums sein? Kern

In sehr hohem Maße 1	2	3	4	Gar nicht 5	
☐	☐	☐	☐	☐	Vorbereitung auf den Umgang mit englischsprachiger Literatur
☐	☐	☐	☐	☐	Vorbereitung auf englischsprachige Fachkommunikation

Anhang 1 zu Giesler M, Huber J, Paulmann V. Quality assurance and curricular development of medical faculties using graduate surveys: Challanges - proposal of care questionnaire - implemention guide. GMS J Med Educ. 2022;39(1):Doc10. DOI: 10.3205/zma001531

☐	☐	☐	☐	☐	Angebote zum Erwerb wissenschaftlicher Arbeitsweisen
☐	☐	☐	☐	☐	Training von mündlicher Präsentation
☐	☐	☐	☐	☐	Angebote zum Verfassen von wissenschaftlichen Texten
☐	☐	☐	☐	☐	Fachliche Vertiefungsmöglichkeiten (Wahlfach)
☐	☐	☐	☐	☐	Forschungsbezug von Lehre und Lernen (Umformulieren)
☐	☐	☐	☐	☐	Betreuung durch Lehrende
☐	☐	☐	☐	☐	Angebote zum Erwerb von Lernstrategien
☐	☐	☐	☐	☐	Training zur Weitergabe von Informationen / Fachwissen an Patienten
☐	☐	☐	☐	☐	Training zur Weitergabe von Informationen / Fachwissen an Mitglieder anderer medizinischer Berufsgruppen *(z.B. Pflegekräfte, ...)*
☐	☐	☐	☐	☐	Angebote zum Erwerb kommunikativer Fertigkeiten
☐	☐	☐	☐	☐	Angebote zum Erwerb von Selbstmanagement-Fertigkeiten wie z. B. Zielsetzung, Selbstinstruktion, Selbstverstärkung
☐	☐	☐	☐	☐	Angebote zum Umgang mit ethischen Fragen in der Berufspraxis
☐	☐	☐	☐	☐	Einsatz moderner Lehrformen (z.B. POL, Skills-Lab, Rollenspiele mit Simulationspatienten)
☐	☐	☐	☐	☐	Erkennbarkeit von Lehr- und Lernzielen in den Fachgebieten
☐	☐	☐	☐	☐	Inhaltliche Abstimmung zwischen den Lehrveranstaltungen

24 Wie beurteilen Sie die folgenden praxis- und berufsbezogenen Elemente in Ihrem Fach? Kern

Sehr gut				Sehr schlecht	
1	2	3	4	5	
☐	☐	☐	☐	☐	Aktualität der vermittelten Lehrinhalte bezogen auf Praxisanforderungen
☐	☐	☐	☐	☐	Verknüpfung von Theorie und Praxis
☐	☐	☐	☐	☐	Vorbereitung auf den Beruf
☐	☐	☐	☐	☐	Unterstützung bei der Stellensuche
☐	☐	☐	☐	☐	Angebot berufsorientierender Veranstaltungen
☐	☐	☐	☐	☐	Unterstützung bei der Suche geeigneter Praktikumsplätze
☐	☐	☐	☐	☐	Lehrende aus der Praxis
☐	☐	☐	☐	☐	Praxisbezogene Lehrinhalte
☐	☐	☐	☐	☐	Pflichtpraktika/Praxissemester
☐	☐	☐	☐	☐	Angebote zum Erwerb von Schlüsselkompetenzen

25 Wie bewerten Sie die Ausbildungsqualität in Ihrem Praktischen Jahr in Bezug auf den Erwerb spezifischer ärztlicher Fähigkeiten?Kern

Sehr gut				Sehr schlecht	
1	2	3	4	5	
☐	☐	☐	☐	☐	Erwerb ärztlicher Fähigkeiten der Inneren Medizin
☐	☐	☐	☐	☐	Erwerb ärztlicher Fähigkeiten der Chirurgie
☐	☐	☐	☐	☐	Erwerb ärztlicher Fähigkeiten im Wahlfach:

26 Wie zufrieden sind Sie aus heutiger Sicht mit Ihrem Studium insgesamt? Kern

Sehr zufrieden				Sehr unzufrieden
1	2	3	4	5
☐	☐	☐	☐	☐

Anhang 1 zu Giesler M, Huber J, Paulmann V. Quality assurance and curricular development of medical faculties using graduate surveys: Challanges - proposal of care questionnaire - implemention guide. GMS J Med Educ. 2022;39(1):Doc10. DOI: 10.3205/zma001531

27	Wenn Sie rückblickend noch einmal die freie Wahl hätten, würden Sie… OPTIONAL

Sehr wahr-scheinlich 1	2	3	4	Sehr unwahr-scheinlich 5	
☐	☐	☐	☐	☐	… denselben Studiengang wählen?
☐	☐	☐	☐	☐	… dieselbe Hochschule wählen?
☐	☐	☐	☐	☐	… wieder studieren?

E Die Situation nach Studienabschluss

28 In welchem Maße verfügten Sie zum Zeitpunkt des Studienabschlusses über die folgenden Fähigkeiten/Kompetenzen? Kern

1 In sehr hohem Maße	2 Hoch	3	4 Gering	5 Gar nicht	
☐	☐	☐	☐	☐	Fähigkeit, Strategien zur Problemlösung zu entwickeln
☐	☐	☐	☐	☐	Fähigkeit, zu planen und zu organisieren
☐	☐	☐	☐	☐	Fähigkeit, sich mündlich adäquat auszudrücken
☐	☐	☐	☐	☐	Fähigkeit, sich schriftlich adäquat auszudrücken
☐	☐	☐	☐	☐	Fähigkeit, auf Englisch zu kommunizieren
☐	☐	☐	☐	☐	Fähigkeit, sich selbst zu reflektieren
☐	☐	☐	☐	☐	Fähigkeit, mit empfangener Kritik umzugehen
☐	☐	☐	☐	☐	Fähigkeit, konstruktiv Kritik zu üben
☐	☐	☐	☐	☐	Fähigkeit, im Team zusammenzuarbeiten
☐	☐	☐	☐	☐	Fähigkeit, theoretisches Wissen praktisch anzuwenden
☐	☐	☐	☐	☐	Fähigkeit, sich neuen Situationen flexibel anzupassen
☐	☐	☐	☐	☐	Fähigkeit, neue Ideen zu entwickeln
☐	☐	☐	☐	☐	Fähigkeit, eigenständig zu arbeiten
☐	☐	☐	☐	☐	Beherrschung des eigenen Faches, der eigenen Disziplin

29 In welchem Maße verfügten Sie bei Studienabschluss über die folgenden medizinbezogenen Kompetenzen? Kern

Der Freiburger Fragebogen zur Erfassung von Kompetenzen in der Medizin (FKM) (Giesler, Forster, Biller, Fabry 2011) kann in Freiburg beim Studiendekanat der Medizinischen Fakultät / Kompetenzzentrum Evaluation BW angefordert werden.

F BESCHÄFTIGUNGSSUCHE

**30**	**Haben Sie eine Beschäftigung im Bereich der Krankenversorgung gesucht?** Kern
☐	Ja
☐	Nein

31 Welche Rolle spielten folgende Gründe für Ihre Stellenwahl? Kern

**1-Sehr große Rolle**	**2-Große Rolle**	**3**	**4-kleine Rolle**	**5-Gar keine Rolle**	
☐	☐	☐	☐	☐	Fachliches Interesse
☐	☐	☐	☐	☐	Reputation des Arbeitgebers
☐	☐	☐	☐	☐	Allgemeine Arbeitsbedingungen
☐	☐	☐	☐	☐	Verdienstmöglichkeiten
☐	☐	☐	☐	☐	Familienfreundlichkeit des Arbeitgebers
☐	☐	☐	☐	☐	Gute Weiterbildungsangebote
☐	☐	☐	☐	☐	Gute Möglichkeit zur fachlichen Qualifikation
☐	☐	☐	☐	☐	Partner/Partnerin in derselben Stadt
☐	☐	☐	☐	☐	Gute berufliche Aufstiegsmöglichkeiten
☐	☐	☐	☐	☐	Nähe zum bisherigen Wohnort
☐	☐	☐	☐	☐	Familiäre Gründe
☐	☐	☐	☐	☐	Positive Arbeitserfahrung bei dem Arbeitgeber
☐	☐	☐	☐	☐	Gute Arbeitszeitenregelung
☐	☐	☐	☐	☐	Sonstiges

32 Wie haben Sie nach Studienabschluss versucht eine Stelle zu finden? Optional

☐	Bewerbung auf ausgeschriebene Stellen (z.B. Zeitung, Internet, Aushang)
☐	Eigenständige Kontaktaufnahme zu Arbeitgebern (Blindbewerbung/Initiativbewerbung)
☐	Besuch von Firmenkontaktmessen
☐	Durch PJ/Famulatur während des Studiums
☐	Inanspruchnahme von Angeboten der Agentur für Arbeit
☐	Nutzung von webbasierten Netzwerken (z.B. Xing, LinkedIn)
☐	Nutzung von privaten Vermittlungsagenturen
☐	Nutzung der Angebote des Career Service/Career Center
☐	Nutzung andere Angebote zur Stellensuche an der Hochschule (ausgenommen Career Service/Career Center)
☐	Über bereits bestehende Kontakte
☐	Sonstiges:

33 Wie viele Monate hat Ihre Suche nach einer Beschäftigung insgesamt gedauert? Optional

Monate

34 Welche Vorgehensweise führte zu Ihrer ersten Beschäftigung nach Studienabschluss? Optional

☐	Bewerbung auf ausgeschriebene Stellen (z.B. Zeitung, Internet, Aushang)
☐	Eigenständige Kontaktaufnahme zu Arbeitgebern (Blindbewerbung/Initiativbewerbung)
☐	Besuch von Firmenkontaktmessen
☐	Durch PJ/Famulatur während des Studiums
☐	Inanspruchnahme von Angeboten der Agentur für Arbeit
☐	Nutzung von webbasierten Netzwerken (z.B. Xing, LinkedIn)
☐	Nutzung von privaten Vermittlungsagenturen

☐	Nutzung der Angebote des Career Service/Career Center
☐	Nutzung andere Angebote zur Stellensuche an der Hochschule (ausgenommen Career Service/Career Center)
☐	Über bereits bestehende Kontakte
☐	Sonstiges:

G DERZEITIGE TÄTIGKEIT UND BESCHÄFTIGUNGSSITUATION

35 Was trifft auf ihre derzeitige Situation zu? (Mehrfachnennungen möglich) Kern

☐	Ich übe eine ärztliche Tätigkeit innerhalb der Krankenversorgung aus	
		☐ Arztpraxis
		☐ Zahnarztpraxis
		☐ Medizinisches Versorgungszentrum
		☐ Universitätsklinikum
		☐ Krankenhaus (nicht Universitätsklinikum)
		☐ Öffentlicher Gesundheitsdienst
		☐ Medizinischer Tätigkeitsbereich in der Privatwirtschaft
		☐ anderer medizinischer Tätigkeitsbereich: ……………………………………………………………………………………
☐	Ich übe eine ärztliche Tätigkeit außerhalb der Krankenversorgung aus (z.B. Beratung, Forschung)	
☐	Ich übe eine nichtärztliche Tätigkeit aus<br>(Betätigungsfeld bitte eintragen):	
☐	Ich arbeite an meiner Dissertation	
☐	Ich bin zurzeit arbeitslos	
☐	Ich habe eine Familienpause eingelegt	
☐	Ich studiere ein anderes Fach	
☐	Ich bin derzeit nicht berufstätig, weil…	

36 Sind Sie derzeit befristet oder unbefristet beschäftigt? Kern

☐	Unbefristet
☐	Befristet

37 Wie viele Stunden arbeiten Sie durchschnittlich pro Woche? Kern

	Vertragswochenarbeitszeit (in Stunden): …………
	Tatsächliche Wochenarbeitszeit (in Stunden):…………xxxxxx

38 Wie hoch ist Ihr monatliches Bruttoeinkommen in Euro (inkl. Sonderzahlungen und Überstunden)? Kern

☐	Bis zu 250 €
☐	251 - 500 €
☐	501 - 750 €
☐	751 - 1000 €
☐	1.001 - 1.250 €
☐	1.251 - 1.500 €
☐	1.501 - 1.750 €
☐	1.751 - 2.000 €
☐	2.001 - 2.250 €

Anhang 1 zu Giesler M, Huber J, Paulmann V. Quality assurance and curricular development of medical faculties using graduate surveys: Challanges - proposal of care questionnaire - implemention guide. GMS J Med Educ. 2022;39(1):Doc10. DOI: 10.3205/zma001531

☐	2.251 - 2.500 €
☐	2.501 - 2.750 €
☐	2.751 - 3.000 €
☐	3.001 - 3.500 €
☐	3.501 - 4.000 €
☐	4.001 - 4.500 €
☐	4.501 - 5.000 €
☐	5.001 - 5.500 €
☐	5.501 - 6.000 €
☐	6.001 - 6.500 €
☐	über 6.500 €
**39**	**In welcher Region sind Sie derzeit beschäftigt** Kern
☐	In Deutschland → bitte geben sie die Postleitzahl an:<br>Falls Postleitzahl nicht bekannt → bitte nennen Sie den Ort
☐	In einem anderen Land *(bitte angeben):* …………………………………………………………………………………………………

40 In welchem Bereich arbeiten Sie derzeit? Kern

☐	Arztpraxis	
☐	Zahnarztpraxis	
☐	Medizinisches Versorgungszentrum	
☐	Universitätsklinikum	
☐	Universität	
☐	Krankenhaus (nicht Universitätsklinikum)	
☐	Öffentlicher Gesundheitsdienst	
☐	Medizinischer Tätigkeitsbereich in der Privatwirtschaft	
☐	Forschung (öffentliche Hand)	
☐	Forschung (Privatwirtschaft	
☐	Anderer medizinischer Tätigkeitsbereich:	
☐	Nichtmedizinischer Tätigkeitsbereich	
	Humanmedizin er mit fachärztliche Weiterbildung: Wenn Sie an Ihre erste Tätigkeit nach dem Studium denken: In welchem Bereich der Medizin, des Gesundheitswesens, der sozialen und gesundheitsbezogenen Dienstleistungen waren/sind Sie tätig?	
		☐ Klinische Medizin mit Grund- und Regelversorgung (Versorgungsstufe I)
		☐ Klinische Medizin, Schwerpunktversorgung (Versorgungsstufe II)
		☐ Klinische Medizin, Universitätsklinik/Maximalversorgung (Versorgungsstufe III)
		☐ Niedergelassener Arzt
		☐ Soziale und gesundheitsbezogene Dienstleistungen
		☐ Sonstiges:
	Humanmedizin er ohne fachärztliche Weiterbildung: Wenn Sie an Ihre erste Tätigkeit nach dem Studium denken: In welchem Bereich waren/sind Sie tätig?	
		☐ außeruniversitäre Forschungseinrichtung
		☐ Privatwirtschaft
		☐ universitäre Forschung
		☐ Klinische Medizin mit Grund- und Regelversorgung (Versorgungsstufe I)
		☐ Klinische Medizin, Schwerpunktversorgung (Versorgungsstufe II)
		☐ Klinische Medizin, Universitätsklinik/Maximalversorgung (Versorgungsstufe III)

ENTWURF

Anhang 1 zu Giesler M, Huber J, Paulmann V. Quality assurance and curricular development of medical faculties using graduate surveys: Challanges - proposal of care questionnaire - implemention guide. GMS J Med Educ. 2022;39(1):Doc10. DOI: 10.3205/zma001531

	☐ Niedergelassener Arzt
	☐ Soziale und gesundheitsbezogene Dienstleistungen
	☐ Stiftung/gemeinnützige Einrichtung o. Ä.
	☐ Sonstiges:

41 Welche fach(zahn)ärztliche Weiterbildung streben Sie an? Kern

- ☐ Nicht zutreffend, ich strebe derzeit keine fachärztliche Weiterbildung an
- ☐ Allgemeinmedizin
- ☐ Anästhesiologie
- ☐ Anatomie
- ☐ Arbeitsmedizin / Sozialmedizin
- ☐ Augenheilkunde
- ☐ Biochemie
- ☐ Chirurgie
- ☐ Dermatologie / Venerologie
- ☐ Gynäkologie und Geburtshilfe
- ☐ Hals-, Nasen- und Ohrenheilkunde
- ☐ Haut- und Geschlechtskrankheiten
- ☐ Humangenetik
- ☐ Innere Medizin
- ☐ Kieferorthopädie
- ☐ Kinder- und Jugendpsychiatrie
- ☐ Kinderheilkunde / Kinder- und Jugendmedizin
- ☐ Klinische Chemie / Laboratoriumsdiagnostik / Laboratoriumsmedizin
- ☐ Klinische Pharmakologie / Pharmakotherapie
- ☐ Mikrobiologie / Virologie
- ☐ Mund-Kiefer-Gesichtschirurgie
- ☐ Neurochirurgie
- ☐ Neurologie
- ☐ Nuklearmedizin / Strahlenbehandlung / Strahlenschutz
- ☐ Öffentliches Gesundheitswesen
- ☐ Oralchirurgie
- ☐ Parodontologie
- ☐ Orthopädie / Unfallchirurgie
- ☐ Pathologie
- ☐ Pharmakologie / Toxikologie
- ☐ Psychiatrie und Psychotherapie
- ☐ Psychosomatik und Psychotherapeutische Medizin
- ☐ Radiologie
- ☐ Rechtsmedizin
- ☐ Umweltmedizin und Hygiene
- ☐ Urologie
- ☐ Sonstiges und zwar: ....

Anhang 1 zu Giesler M, Huber J, Paulmann V. Quality assurance and curricular development of medical faculties using graduate surveys: Challanges - proposal of care questionnaire - implemention guide. GMS J Med Educ. 2022;39(1):Doc10. DOI: 10.3205/zma001531

42	**Inwieweit werden die folgenden medizinbezogenen Kompetenzen in Ihrer gegenwärtigen Erwerbs gefordert? Kern**

Der Freiburger Fragebogen zur Erfassung von Kompetenzen in der Medizin (FKM) (Giesler, Forster, Biller, Fabry 2011) kann in Freiburg beim Studiendekanat der Medizinischen Fakultät / Kompetenzzentrum Evaluation BW angefordert werden.

H ZUM ZUSAMMENHANG VON STUDIUM UND BERUF

43 Wenn Sie alle Aspekte Ihrer beruflichen Situation berücksichtigen: Inwieweit entspricht Ihre derzeitige berufliche Situation den Erwartungen, ... OPTIONAL

In sehr hohem Maße 1	2	3	4	Gar nicht 5	
☐	☐	☐	☐	☐	..die Sie bei Studienbeginn hatten?
☐	☐	☐	☐	☐	…die Sie bei Studienabschluss hatten?

I BERUFLICHE ORIENTIERUNGEN UND ARBEITSZUFRIEDENHEIT

44 Wie wichtig sind Ihnen im Allgemeinen die folgenden Aspekte des Berufs? Kern

Sehr wichtig 1	2	3	4	Gar nicht wichtig 5	
☐	☐	☐	☐	☐	Weitgehend eigenständige Arbeitsplanung
☐	☐	☐	☐	☐	Möglichkeit zur wissenschaftlichen Arbeit
☐	☐	☐	☐	☐	Übersichtliche und geregelte Arbeitsaufgaben
☐	☐	☐	☐	☐	Möglichkeit zur Verwendung erworbener Kompetenzen
☐	☐	☐	☐	☐	Arbeitsplatzsicherheit
☐	☐	☐	☐	☐	Gesellschaftliche Achtung und Anerkennung
☐	☐	☐	☐	☐	Möglichkeit, eigene Ideen zu verwirklichen
☐	☐	☐	☐	☐	Gutes Betriebsklima
☐	☐	☐	☐	☐	Möglichkeit zur beruflichen Weiterqualifizierung
☐	☐	☐	☐	☐	Hohes Einkommen
☐	☐	☐	☐	☐	Möglichkeit zur gesellschaftlichen Einflussnahme
☐	☐	☐	☐	☐	Eine Arbeit zu haben, die mich fordert
☐	☐	☐	☐	☐	Gute Aufstiegsmöglichkeiten
☐	☐	☐	☐	☐	Übernahme von Koordinations- und Leitungsaufgaben
☐	☐	☐	☐	☐	Möglichkeit, Nützliches für die Allgemeinheit zu tun
☐	☐	☐	☐	☐	Gute Möglichkeit, familiäre Aufgaben mit dem Beruf zu vereinbaren
☐	☐	☐	☐	☐	Genug Zeit für Freizeitaktivitäten
☐	☐	☐	☐	☐	Interessante Arbeitsinhalte

Anhang 1 zu Giesler M, Huber J, Paulmann V. Quality assurance and curricular development of medical faculties using graduate surveys: Challanges - proposal of care questionnaire - implemention guide. GMS J Med Educ. 2022;39(1):Doc10. DOI: 10.3205/zma001531

45 In welchem Maße treffen die folgenden Aussagen auf Ihre gegenwärtige berufliche Situation zu? Kern

In sehr hohem Maße 1	2	3	4	Gar nicht 5	
☐	☐	☐	☐	☐	Weitgehend eigenständige Arbeitsplanung
☐	☐	☐	☐	☐	Möglichkeit zur wissenschaftlichen Arbeit
☐	☐	☐	☐	☐	Übersichtliche und geregelte Arbeitsaufgaben
☐	☐	☐	☐	☐	Möglichkeit zur Verwendung erworbener Kompetenzen
☐	☐	☐	☐	☐	Arbeitsplatzsicherheit
☐	☐	☐	☐	☐	Gesellschaftliche Achtung und Anerkennung
☐	☐	☐	☐	☐	Möglichkeit, eigene Ideen zu verwirklichen
☐	☐	☐	☐	☐	Gutes Betriebsklima
☐	☐	☐	☐	☐	Möglichkeit zur beruflichen Weiterqualifizierung
☐	☐	☐	☐	☐	Hohes Einkommen
☐	☐	☐	☐	☐	Möglichkeit zur gesellschaftlichen Einflussnahme
☐	☐	☐	☐	☐	Eine Arbeit zu haben, die mich fordert
☐	☐	☐	☐	☐	Gute Aufstiegsmöglichkeiten
☐	☐	☐	☐	☐	Übernahme von Koordinations- und Leitungsaufgaben
☐	☐	☐	☐	☐	Möglichkeit, Nützliches für die Allgemeinheit zu tun
☐	☐	☐	☐	☐	Gute Möglichkeit, familiäre Aufgaben mit dem Beruf zu vereinbaren
☐	☐	☐	☐	☐	Genug Zeit für Freizeitaktivitäten
☐	☐	☐	☐	☐	Interessante Arbeitsinhalte

J ANGABEN ZUR PERSON

46 Was ist Ihr Geschlecht? Kern

☐ Männlich

☐ Weiblich

☐ Divers

47 In welchem Jahr sind Sie geboren? Kern

☐☐☐☐☐

48 In welchem Land wurden Sie geboren Kern

☐ In Deutschland

☐ In einem anderen Land:

49 Wo wohnen sie derzeit? OPTIONAL

☐ In Deutschland → Bitte geben Sie die Postleitzahl an: Falls Postleitzahl nicht bekannt ist → Bitte nennen Sie den Ort:

☐ In einem anderen Land → Bitte geben Sie den Namen des Landes an:

Anhang 1 zu Giesler M, Huber J, Paulmann V. Quality assurance and curricular development of medical faculties using graduate surveys: Challanges - proposal of care questionnaire - implemention guide. GMS J Med Educ. 2022;39(1):Doc10. DOI: 10.3205/zma001531

50 In welchem Land wurden Ihre Eltern geboren? Kern

- ☐ In Deutschland
- ☐ In einem anderen Land:

51 Welche Staatsangehörigkeit(en) haben Sie? (Mehrfachnennung möglich) Kern

- ☐ Die deutsche Staatsangehörigkeit
- ☐ Eine andere Staatsangehörigkeit

52 Was ist Ihre derzeitige Lebenssituation? Kern

- ☐ Ledig, ohne Partner/in
- ☐ Ledig, mit Partner/in
- ☐ Verheiratet
- ☐ Sonstiges

53 Hatten/Haben Sie Kinder, die mit Ihnen in einem Haushalt leben? Kern

- ☐ Ja → Wie Viele?
- ☐ Nein

54 Wer ist in der Regel an der Betreuung Ihres Kindes/Ihrer Kinder tagsüber beteiligt? (Mehrfachnennung möglich) Kern

- ☐ Ich habe die Betreuung selbst übernommen
- ☐ Mein Partner/meine Partnerin
- ☐ Tagesmutter, Babysitter(in), Au-Pair
- ☐ Kinderkrippe, Kindergarten oder schulische Nachmittagsbetreuung
- ☐ Eltern, andere Verwandte oder Freunde
- ☐ Es ist keine Betreuung notwendig
- ☐ Sonstiges:

55 Haben Sie während des Studiums ein Kind/Kinder bekommen? Kern

- ☐ Ja, im vorklinischen Studienabschnitt
- ☐ Ja, im klinischen Studienabschnitt
- ☐ Ja, im Praktischen Jahr
- ☐ Nein

56 Was ist der höchste berufliche Bildungsabschluss Ihres Partners/Ihrer Partnerin? OPTIONAL

- Lehre/ Berufsausbildung
- Fachschulabschluss
- Fachhochschulabschluss
- Universitätsabschluss
- Promotion
- Habilitation
- Ohne beruflichen Bildungsabschluss

57 Ist er/sie in einem medizinischen/medizinnahen Bereich tätig? OPTIONAL

- Ja
- Nein, in einem anderen Bereich
- Nein, er/sie ist zur Zeit gar nicht berufstätig

ENTWURF

Anhang 1 zu Giesler M, Huber J, Paulmann V. Quality assurance and curricular development of medical faculties using graduate surveys: Challanges - proposal of care questionnaire - implemention guide. GMS J Med Educ. 2022;39(1):Doc10. DOI: 10.3205/zma001531

58 Wie beurteilen Sie rückblickend die Vereinbarkeit von Studium und Kindererziehung? OPTIONAL

Sehr gut				Sehr schlecht
1	2	3	4	5
☐	☐	☐	☐	☐

59 Welchen höchsten beruflichen Abschluss haben Ihre Eltern? single choice jeweils für Vater und Mutter **OPTIONAL**

- Lehre oder Facharbeiterabschluss
- Berufsfach- oder Handelsschulabschluss
- Meisterprüfung oder staatlich geprüfter Techniker
- Abschluss an einer Fachschule (DDR)
- Abschluss an einer Fachhochschule
- Abschluss an einer Ingenieurschule oder Handelsakademie
- Abschluss an einer Kunst- oder Musikhochschule
- Abschluss an einer Universität
- Promotion
- Habilitation
- Keinen beruflichen Abschluss
- Nicht bekannt
- Sonstiges:

60 Welchen Beruf üben bzw. übten Ihre Eltern aus? Wenn nicht mehr berufstätig, bitte den zuletzt ausgeübten Beruf angeben OPTIONAL

offen jeweils für Vater und Mutter

K KOMMENTARE / ANREGUNGEN

Weitere Kommentare und Anregungen zu Ihrer Hochschule / Ihrem Studiengang, die Sie zur Sprache bringen möchten, teilen Sie uns bitte im Folgenden mit.

61 Was hat Ihnen besonders gut an Ihrem Studium gefallen? Kern

....

....

....

....

....

....

....

....

62 Welche Aspekte im Studium haben besonders dazu beigetragen, dass Sie sich gut auf Ihren Beruf vorbereitet gefühlt haben? OPTIONAL

....

....

....

....

ENTWURF

Anhang 1 zu Giesler M, Huber J, Paulmann V. Quality assurance and curricular development of medical faculties using graduate surveys: Challanges - proposal of care questionnaire - implemention guide. GMS J Med Educ. 2022;39(1):Doc10. DOI: 10.3205/zma001531

……

……

……

……

63 **Welche Aspekte im Studium empfehlen Sie zu verändern, so dass künftige Ärzte noch besser auf ihren Beruf vorbereitet sind?** OPTIONAL

……

……

……

……

……

……

……

……

64 **HumanmedizinerInnen: Welche Aspekte im PJ haben besonders dazu beigetragen, dass Sie sich gut auf Ihren Beruf vorbereitet gefühlt haben?** OPTIONAL

……

……

……

……

……

……

……

……

65 **HumanmedizinerInnen: Welche Aspekte im PJ empfehlen Sie zu verändern, so dass künftige Ärzte noch besser auf ihren Beruf vorbereitet sind?** OPTIONAL

……

……

……

……

……

……

Anhang 1 zu Giesler M, Huber J, Paulmann V. Quality assurance and curricular development of medical faculties using graduate surveys: Challanges - proposal of care questionnaire - implemention guide. GMS J Med Educ. 2022;39(1):Doc10. DOI: 10.3205/zma001531

66 66 **Welche wichtigen Veränderungen sollten Ihrer Ansicht nach an Ihrer Hochschule / in Ihrem Studiengang erfolgen?**

....

....

....

....

....

....

....

....

ENTWURF

Anhang 1 zu Giesler M, Huber J, Paulmann V. Quality assurance and curricular development of medical faculties using graduate surveys: Challanges - proposal of care questionnaire - implemention guide. GMS J Med Educ. 2022;39(1):Doc10. DOI: 10.3205/zma001531

L KONTAKTE ZU IHRER UNIVERSITÄT

Wir bitten Sie, uns für den Fall, dass Sie sich an der Nachfolgestudie beteiligen (A) möchten, Ihre Adresse mitzuteilen. Die Adresse werden wir selbstverständlich getrennt von den Fragebogendaten elektronisch speichern und nach den Bestimmungen des Datenschutzes ausschließlich für den angegebenen Zweck verwenden.

Diese Seite des Fragebogens mit Ihrer Adresse wird aus Datenschutzgründen sofort nach Eingang des Fragebogens getrennt aufbewahrt.

A Würden Sie sich auch an einer Nachfolgestudie zum Berufsverlauf beteiligen, die wir in ca. 3-4 Jahren durchführen wollen?

- ☐ Ja
- ☐ Nein

Anschrift:

Name: ____

Straße, Hausnummer: ____

Postleitzahl, Ort: ____

ODER:

E-Mail-Adresse: ____

Ich willige darin ein, dass meine Kontaktdaten für die Zusendung des Ergebnisberichts und/oder eine weitere Befragung in drei bis vier Jahren von der Universität Freiburg verarbeitet werden. Mir ist bekannt, dass ich die Einwilligung jederzeit widerrufen kann, die Rechtmäßigkeit der aufgrund der Einwilligung bis zum Widerruf erfolgten Verarbeitung allerdings nicht berührt wird. Ich habe verstanden, dass ich mich für einen Widerruf einfach an folgende Kontaktperson werden kann xxxxxxx (xxxxx@uniklinikxxxxx.de)

____

Datum, Unterschrift

Sie haben das Recht, bei der xxxxx Auskunft über die zu Ihre Person gespeicherten Daten zu verlangen sowie unrichtige Daten berichtigen zu lassen. Ein entsprechendes Ersuchen richten Sie bitte xxxxxxx (xxxxxxx@uniklinik-xxxxxx.de)

Anhang 1 zu Giesler M, Huber J, Paulmann V. Quality assurance and curricular development of medical faculties using graduate surveys: Challanges - proposal of care questionnaire - implemention guide. GMS J Med Educ. 2022;39(1):Doc10. DOI: 10.3205/zma001531
