# Supplementary material for: Quality assurance and curricular development of medical faculties using graduate surveys: Challenges – proposal of a core questionnaire – implementation guide
Source: GMS J Med Educ. 2022 Feb 15;39(1):Doc10. doi: 10.3205/zma001531 (PMC8953197; doi:10.3205/zma001531)
Supplement: Procedural elements of graduate surveys [file JME-39-10-s-002.pdf]

Procedural elements of graduate surveys				
**Topic**	**Description**	**Options**	**Problems**	**Source**
**Address search**	**How are addresses updated?**<br><br>Good research is central to the success of the survey.	• Postal mail: last known contact from time as a student (student admissions office, student affairs office, etc.), parents' address, if available.<br>• Research via the government registry offices (partially free of charge; § 8 VwVfG: As public institutions, universities may request addresses within the scope of administrative assistance); Advance purging of outdated addresses to improve address quality.	The professional mobility of physicians is relatively high: It is often difficult to contact students after graduation (moves, student email addresses are deactivated, private email addresses fall under data protection, etc.).	§ 8 Verwaltungsverfahrensgesetz (VwVfG)
**Alumni**	**Should the alumni association be involved?**<br><br>Alumni associations often maintain good contact with graduates.	• Mailing campaigns are done through the alumni associations which have current address lists.	• Selection bias: Not all graduates join an alumni association. Generally, it is those who have positive attitudes toward the university.<br>• Contact is only possible if there has been consent to have address information shared for other purposes than those of the alumni association.	GDPR (General Data Protection Regulation)
**Letter for mailing**	**How should the letter be written and what should it contain?**<br><br>A letter should contain the most important information and have a motivational effect.	• Letter is written by a university official (president, dean of studies) to underscore the importance of the survey.<br>• The signature of an academic dean or university professor can through familiarity lead to a stronger identification with the medical school and thus to a higher level of participation.<br>• Personally addressing the letter with the proper title and surname.<br>• Contact leads to the highest response rates.<br>• Email messages with a direct personalized link to the survey page is recommended for online surveys, more so than a TAN or password login).		• Bortz J, Döring N. Forschungsmethoden und Evaluation. 4th ed. Heidelberg: Springer. 2006.<br>• Phillips AW, Reddy S, Durning SJ. Improving response rates and evaluating nonresponse bias in surveys: AMEE Guide No. 102. Med Teach. 2016;38:217-228.<br>• Saleh A, Bista K. Examining Factors Impacting Online Survey Response Rates in Educational Research: Perceptions of Graduate Students. Journal of Multi-Disciplinary Evaluation. 2017;13(29):63-74.<br>• Scherer C, Schmidt S. Integration der Absolventenbefragung in die

Anhang 2 zu Giesler M, Huber J, Paulmann V. Quality assurance and curricular development of medical faculties using graduate surveys: Challanges - proposal of care questionnaire - implemention guide. GMS J Med Educ. 2022;39(1):Doc10. DOI: 10.3205/zma001531

Procedural elements of graduate surveys				
**Topic**	**Description**	**Options**	**Problems**	**Source**
				Qualitätssicherung von Studium und Lehre an der Goethe Universität Frankfurt am Main. In: Falk S, Reimer M, Hrsg. Absolventenstudien und Qualitätsmanagement: Best practice an deutschen und österreichischen Hochschulen. Münster: Waxmann; 2018. p. 63-78.<br>• Van Mol. Improving web survey efficiency: the impact of an extra reminder and reminder content on web survey response. International Journal of Social Research Methodology. 2016;20(4):317-327.
**Type of survey/ method**	**Which method should be used for the survey?**<br><br>Surveys can be online or paper-based. A mixed-methods approach is also possible.	•Data from online surveys are more quickly available and contain fewer errors<br>• Responsive design is recommended (=automatic adaptation to the device used)<br>• Increased response rate through the additional use of paper-based questionnaires.<br>• Mixed design of online and paper-based questionnaires has been proven to serve different preferences and enable additional contacts (experience with graduate surveys in medicine).	• Errors can occur when entering data from paper-based questionnaires. The entries also need to be checked when scanning them.<br>• Staff and material costs for a paper-based questionnaire with mailing are higher than for an online survey.<br>• Physicians have less time and opportunity to fill out a questionnaire online during working hours. It is easier to fill out a paper-based questionnaire while on night duty.<br>• Be sure to use identical letters and questionnaire formats when following a mixed-methods approach to prevent method effects as a result of different modi.	• Maurer M, Jandura O. Masse statt Klasse? Einige kritische Anmerkungen zu Repräsentativität und Validität von Online-Befragungen. In: Jackob N, Schoen H, Zerback T, Hrsg. Sozialforschung im Internet. Wiesbaden: VS Verlag für Sozialwissenschaften; 2009.<br>• Phillips AW, Reddy S, Durning SJ. Improving response rates and evaluating nonresponse bias in surveys: AMEE Guide No. 102. Med Teach. 2016;38:217-228.<br>• Zentrum für Umfragen, Methoden und Analysen -ZUMA-. 2014. Available from: https://nbn-resolving.org/urn:nbn:de:0168-ssoar-201415

Anhang 2 zu Giesler M, Huber J, Paulmann V. Quality assurance and curricular development of medical faculties using graduate surveys: Challanges - proposal of care questionnaire - implemention guide. GMS J Med Educ. 2022;39(1):Doc10. DOI: 10.3205/zma001531

Procedural elements of graduate surveys				
**Topic**	**Description**	**Options**	**Problems**	**Source**
**Benchmark**	**How to receive comparative data from other universities?**<br><br>Some results can be difficult to interpret without the opportunity to compare data. Comparative data can sharpen the view of the medical school's profile and the strengths and weaknesses of the study program.	• Enter into collaborations/build networks (at the state level or beyond) while using a questionnaire that is as uniform as possible.<br>• The analyses can be performed separately for each university or for the network (anonymized or with the real names of the participating universities).	• Collaborations: Consent to non-anonymized comparisons does not often exist.<br>• Comparisons can be limited by distortions or biases, for instance if different conditions are not taken sufficiently into account.<br>• Small differences in the formulation of questions can influence the responses and limit comparisons.	• Flöther C, Krücken G, Hrsg. Generation Hochschulabschluss: Vielfältige Perspektiven auf Studium und Berufseinstieg. Analysen aus der Absolventenforschung. Münster: Waxmann-Verlag; 2015.<br>• van den Bussche H, Wegscheider K, Zimmermann T. Der Ausbildungserfolg im Vergleich (II). Dtsch Arztebl 2006;103(34–35):A 2225–8.<br>• van den Bussche H, Wegscheider K, Zimmermann T. Der Ausbildungserfolg im Vergleich (III). Dtsch Arztebl 2006;103(47):A 3170–6<br>• Zimmermann T, Wegscheider K, van den Bussche H. Der Ausbildungserfolg im Vergleich (I). Dtsch Arztebl 2006;103(25):A 1732–8.
**Data protection**	**What needs to be taken into account regarding data protection?**<br><br>Who may be contacted? How must addresses and the survey data be kept? Who may be granted access to the data?, etc.<br>The requirements for data protections have increased in recent years. This involves not only the inclusion of a data protection officer prior to conducting a survey and documentation of data processing (GDPR), but also the application for ethical approval.	To be noted:<br><br>• German version of the General Data Protection Regulation (GDPR): https://dsgvo-gesetz.de/<br><br>• Federal Data Protection Act https://www.gesetze-im-internet.de/bdsg_2018/<br><br>• State laws on data protection https://www.audatis.de/ratgeber/lexikon/aufsichtsbehoerden-landesdatenschutzgesetze/	• Taking data protection into account means planning for advance organization.<br>• The answers to some questions cannot be collected as desired due to data protection.	GDPR, Federal Data Protection Act and state laws on data protection

Anhang 2 zu Giesler M, Huber J, Paulmann V. Quality assurance and curricular development of medical faculties using graduate surveys: Challanges - proposal of care questionnaire - implemention guide. GMS J Med Educ. 2022;39(1):Doc10. DOI: 10.3205/zma001531

Procedural elements of graduate surveys				
**Topic**	**Description**	**Options**	**Problems**	**Source**
**Reporting results**	**Which data should be reported?**<br><br>For whom are the data important? How often and in which form should the results be presented (tables, graphs, text, etc.)? The results can be relevant for the medical school curriculum development to check or test the effectiveness of changes. Research questions are also worked on based on graduate survey results. Graduate surveys and regular result reporting also represent an important building block for accreditation procedures.	• A report that complies with data protection rules should be published: This creates publicity for graduate surveys and also serves as a sign for those surveyed that the data are actually used.<br>• The internal reports can be prepared selectively or as an automated full report in the form of tables (if applicable, with graphics).<br>• Alternative: reports written in prose.<br>• The reports can be published annually or according to another interval (as needed).	• Preparing reports takes up a lot of time and resources.<br>• Depending on the target group, reports may need to contain additional information or be presented in shortened versions.<br>• Fatigue effects can emerge if there is frequent repetition of the reports; changes become nearly invisible and can go unperceived.<br>• Data floods make it difficult to see the central developments that are important to the medical school.	• Falk S, Reimer M. Hrsg. Absolventenstudien und Qualitätsmanagement: Best practices an deutschen und östereichischen Hochschulen. Münster: Waxmann; 2018.<br>• Flöther C, Krücken G, Hrsg. Generation Hochschulabschluss: Vielfältige Perspektiven auf Studium und Berufseinstieg. Analysen aus der Absolventenforschung. Münster; 2015.<br>• Janson K. Absolventenstudien. Ihre Bedeutung für die Hochschulentwicklung. Eine empirische Betrachtung. Münster: Waxmann; 2014.
**Reminders**	**How often should graduates be written to?**<br><br>Reminders are necessary to increase response rates.	• Generally, three reminders are recommended: The response rate increases with each reminder (experience with graduate surveys in medicine).	• Each reminder increases the cost (postage, staff): Take note of the cost-benefit ratio.	• Porst, R. Wie man die Rücklaufquote bei postalischen Befragungen erhöht. Mannheim: ZUMA How-to-Reihe, Nr. 09; 2001.<br>• Saleh A, Bista K. Examining Factors Impacting Online Survey Response Rates in Educational Research: Perceptions of Graduate Students. Journal of MultiDisciplinary Evaluation. 2017;13(29):63-74.<br>• Van Mol. Improving web survey efficiency: the impact of an extra reminder and reminder content on web survey response. International Journal of Social Research Methodology. 2016;20(4):317-327.

Anhang 2 zu Giesler M, Huber J, Paulmann V. Quality assurance and curricular development of medical faculties using graduate surveys: Challanges - proposal of care questionnaire - implemention guide. GMS J Med Educ. 2022;39(1):Doc10. DOI: 10.3205/zma001531

Procedural elements of graduate surveys				
**Topic**	**Description**	**Options**	**Problems**	**Source**
**Ethical approval**	**Is an ethical approval needed for the graduate survey?**<br><br>Currently there is no obligation in Germany to obtain ethical approval. However, receiving ethical approval for student and graduate surveys complies with the international standards of Good Research Practice.	• Ethical approval is required if the results are published internationally. Ethical approval is required for some German-language publications if genetic, biometric, or so-called health data are reported (GDPR, Art. 4 (13-15))	• There is no uniform standard to date in Germany for applying for the ethical approval of student/graduate surveys.	GDPR (General Data Protection Regulation)
**Questionnaire**	**Which questions should be asked? How extensive can the questionnaire be?**<br><br>Generally, the blueprint of the questionnaire is the central building block for graduate surveys. There are many recommendations about the aspects to be decided upon. The content of the questionnaire varies from survey to survey depending on the interest/research focus of the stakeholder requesting the survey.	Usually, studies regarding graduates focus on the following:<br>• Employment prior to university study<br>• Course of study / Information about study program<br>• Study conditions and acquisition of skills<br>• Situation after graduating / Job search<br>• Information about current employment / Work situation<br>• Assessment of how university study and profession are connected<br>• Career path<br>• Professional focus and work satisfaction<br>• Personal data<br>• Comments / Suggestions	• Generally, conflicts between research interests and a university's need for information cannot be ruled out. The result can be a disorganized abundance of questions.<br>• Longer questionnaires can result in lower response rates; the risk of dropouts rises	• Janson K. Absolventenstudien. Ihre Bedeutung für die Hochschulentwicklung. Eine empirische Betrachtung. Münster: Waxmann; 2014.<br>• Phillips AW, Reddy S, Durning SJ. Improving response rates and evaluating nonresponse bias in surveys: AMEE Guide No. 102. Med Teach. 2016;38:217-228.<br>• Maurer M, Jandura O. Masse statt Klasse? Einige kritische Anmerkungen zu Repräsentativität und Validität von Online-Befragungen. In: Jackob N, Schoen H, Zerback T, Hrsg. Sozialforschung im Internet. Wiesbaden: VS Verlag für Sozialwissenschaften; 2009.<br>• Schomburg H & Teichler U. Studium, Studienbedingungen und Berufserfolg. In: Teichler U, Daniel H & Enders J, Hrsg. Brennpunkt Hochschule. Neuere Analysen zu Hochschule, Beruf und Gesellschaft. Frankfurt a.M./New York: Springer; 1998. p. 141-172.<br>• Van Mol C. Improving web survey

Anhang 2 zu Giesler M, Huber J, Paulmann V. Quality assurance and curricular development of medical faculties using graduate surveys: Challanges - proposal of care questionnaire - implemention guide. GMS J Med Educ. 2022;39(1):Doc10. DOI: 10.3205/zma001531

Procedural elements of graduate surveys				
**Topic**	**Description**	**Options**	**Problems**	**Source**
				efficiency: the impact of an extra reminder and reminder content on web survey response. International Journal of Social Research Methodology. 2006;20(4):317-327.
**Population**	**Who belongs to my target group?**<br><br>Which time period of graduation do I include? What is the criterion for "graduation" (date on diploma, date of last exam)? How do I find the corresponding graduates (university student office, state examination office, medical associations)?	• Graduation according to academic year<br>• Selected time period for cohorts (e.g., graduation between 2012-2015)<br>• Successfully passing the M3 exam as criterion (information from the state exam offices)<br>• Licensed physicians in the medical associations	• University statistics can have gaps if data sharing with the state examination offices does not function or if the university's own statistical data has errors.	

Anhang 2 zu Giesler M, Huber J, Paulmann V. Quality assurance and curricular development of medical faculties using graduate surveys: Challanges - proposal of care questionnaire - implemention guide. GMS J Med Educ. 2022;39(1):Doc10. DOI: 10.3205/zma001531

Procedural elements of graduate surveys				
**Topic**	**Description**	**Options**	**Problems**	**Source**
**Incentives**	**What effects does the use of incentives have?**<br><br>Ideally, the subjective benefits for the respondents (interesting topic, opinion matters) and the subjective costs (time, disclosure of information) are balanced in a survey . Providing incentives should help to initiate a "socially minded process of sharing." The provision of money as unconditional "compensation" increases willingness to participate. Depending on the situation, the amount will be contingent on the target group and can increase the speed of response.<br>A lottery is not as effective as unconditionally paying out money; ballpoint pens, lapel pins and stamps are also weaker than monetary incentives.	• Money for each participation<br>• Raffling off money or valuable objects among the participants who have filled out a questionnaire<br>• Incentives in the form of university souvenirs (promotional gifts such as ballpoint pens, etc.)	• Ethical attitudes toward "purchased" votes finds them questionable; risk of sample bias.<br>• "Inappropriately lavish" incentives can have the opposite effect and undermine the credibility of the study<br>• Too small an incentive--just like ones that are too large--can upset the balance of the exchange relationship.	• Berger F. Zur Wirkung unterschiedlicher materieller Incentives in postalischen Befragungen: ein Literaturbericht. ZUMA-Nachrichten. 2006;30(58):81-100.<br>• Bortz J, Döring N. Forschungsmethoden und Evaluation. 4th ed. Heidelberg: Springer: Heidelberg; 2006.<br>• Phillips AW, Reddy S, Durning SJ. Improving response rates and evaluating nonresponse bias in surveys: AMEE Guide No. 102. Med Teach. 2016;38:217-228.<br>• Stadtmüller S, Porst R. Zum Einsatz von Incentives bei postalischen Befragungen. Mannheim: ZUMA How-to-Reihe, Nr. 14; 2005.
**Collaboration**	**Who carries out studies on graduates? WIth whom can I cooperate?**<br><br>In Germany there are different regional and national networks that act as "players" in the area of graduate surveys. Advantages of collaborations are the synergetic effects and cost savings that come with a centralized organization (e.g., creating and hosting online questionnaires).	• DZHW (quarterly) – KOAB/ISTAT (annually) – INCHER<br>• Regional: IHF (BAP); KfBH (Saxony); ZQ (RLP)<br>• University networks (e.g., BW - MERLIN/Kompetenznetz enables greater autonomy (Baden-Württemberg (BW) has access to all data in BW and carries out comparative analyses)	• In the case of external partners there are usually contractual obligations that limit flexibility: Changes to the questionnaire are, in part, not possible.<br>• Data and survey are hosted on external servers making access to raw data sometimes limited.	

Anhang 2 zu Giesler M, Huber J, Paulmann V. Quality assurance and curricular development of medical faculties using graduate surveys: Challanges - proposal of care questionnaire - implemention guide. GMS J Med Educ. 2022;39(1):Doc10. DOI: 10.3205/zma001531

Procedural elements of graduate surveys				
**Topic**	**Description**	**Options**	**Problems**	**Source**
**Public relations**	**How can knowledge about the survey be improved? How can the survey be appropriately announced internally and externally? How do I reach potential participants in advance?**	• Point out the importance of survey results early on during university study (e.g., through announcements by the dean of studies to impart the importance to students).<br>• Maintain a website on how graduates are studied to increase visibility.<br>• Include the university's press office to announce the survey in advance.<br>• Networking and maintaining contacts in advance can increase the response rate because participants feel personally included and valued.	• Human resources must be allotted for maintaining contact.	
**Panel surveys**	**How is a graduate survey at multiple time points organized?**	• Obtain consent for additional surveys with the first questionnaire.<br>• Include the medical association or other partners.	• Problem of panel turnover may be more prominent in the case of mobile or very stressed professional groups.<br>• Data protection strategy must be drawn up prior to the first survey time point.<br>• Financing should be ensured over a longer period of time (up to 10 years).	
**Human resources**	**What human resources need to be planned? How should the position requirements be formulated for staff members? How is the workload distributed over the year?**	• Organization/Address search = administrative assistant<br>• Mailing campaign = student assistant<br>• Checking TANs = student assistant<br>• Analyses = academic staff member (50%) and student assistant. Time set at half a year: Student assistant approximately 20 h/month, administrative assistant approximately 12 h/month, analysis approximately 5 h/month.	• Workload is not distributed equally over the year.	
**Represen-tativity**	**How is the representativity of the data verified?**	• Comparison with data from the student admissions office, examination office, etc. (age, sex, scores on state exams).<br>• Check the percentages of admission groups (normal selection process, top of class at second-	• It is often difficult to assess the point in time at which data became biased or distorted.<br>• Corrective procedures are usually not applied.	• Phillips AW, Reddy S, Durning SJ. Improving response rates and evaluating nonresponse bias in surveys: AMEE Guide No. 102. Med Teach. 2016;38:217-228.

Anhang 2 zu Giesler M, Huber J, Paulmann V. Quality assurance and curricular development of medical faculties using graduate surveys: Challanges - proposal of care questionnaire - implemention guide. GMS J Med Educ. 2022;39(1):Doc10. DOI: 10.3205/zma001531

Procedural elements of graduate surveys				
**Topic**	**Description**	**Options**	**Problems**	**Source**
		ary school, waiting list, etc.)<br>• Compare with data from the Federal Office of Statistics or the socioeconomic panel (migrant background, marital status, income)<br>• Compare with examination data IMPP (scores on the state exam)<br>• Compare with data from the state examination office (scores on the state exam)		
**Response rate**	**How is the response rate calculated?**	• The response rate is calculated based on the entire cohort, reagrdless if they were reachable or not (=scientific standard to compare responses independent of the selected method (mail, email, telephone, etc.).<br>• The response rate is calculated based on the actual cases that were reached (only those who were contactable. This better represents the actual willingness to participate).	• In the case of emails, it is hardly possible to determine the number of people actually reached; in the case of traditional mailing campaigns, the letters returned as "undeliverable" are a pre-requisite (defective) for determining unreachability.	• Bortz J, Döring N. Forschungsmethoden und Evaluation. 4th ed. Heidelberg: Springer; 2006.<br>• Multrus F, Majer S. Methodenbericht zum 13. Studierendensurvey. Vergleich Papier-Onlinebefragung. Werkstattbericht. Hefte zur Bildungs- und Hochschulforschung (95). Arbeitsgruppe Hochschulforschung (Hrsg.). Konstanz. 2017.<br>• Phillips AW, Reddy S, Durning SJ. Improving response rates and evaluating nonresponse bias in surveys: AMEE Guide No. 102. Med Teach. 2016;38:217-228.<br>• Porst, R. Wie man die Rücklaufquote bei postalischen Befragungen erhöht. GESIS-How-to, 9. Zentrum für Umfragen, Methoden und Analysen. 2001. Available from: https://nbn-resolving.org/urn:nbn:de:0168-ssoar-201415<br>• Ramm, M. Response, Stichprobe und Repräsentativität. Zwei Dokumentationen zum Deutschen Studie-

Anhang 2 zu Giesler M, Huber J, Paulmann V. Quality assurance and curricular development of medical faculties using graduate surveys: Challanges - proposal of care questionnaire - implemention guide. GMS J Med Educ. 2022;39(1):Doc10. DOI: 10.3205/zma001531

Procedural elements of graduate surveys				
**Topic**	**Description**	**Options**	**Problems**	**Source**
				rendensurvey (DSS). Hefte zur Bil-dungs-und Hochschulforschung (72). Arbeitsgruppe Hochschulforschung (Hrsg.). Universität Konstanz; 2014.
**Survey management**	**What is the most logical place for or-ganizing a graduate survey?**<br><br>Study program level, department level, university level, external cooperative partners /service providers?	• University-wide coordination. Advantages: syn-ergetic effects through centralized organization; internal comparability of the responses is possi-ble.<br>• Internal coordination for the medical school or study program. Advantages: higher motivation as a result of decentralized planning and greater familiarity with the particular conditions (subject knowledge).<br>• External coordination (service provider); mailing campaign / establishing contact through universi-ty. Advantages: professional management of the field work;	• In the case of centralized, university-wide organization, there is less oppor-tunity to influence the details and possi-bly tight resources.<br>• Medical school or study program level: time consuming and work intensive (see point above for "Human resources").<br>• In the case of external coordination, there is possibly less influence on the details regarding how the survey is con-ducted.	
**Time point / Time period**	**When should a graduate survey be conducted?**	• 1.5 to 2 years after graduation to cover both the retrospective view of university study and the career entry phase (clinical experiences, post-graduate education goals).<br>• Survey time period (duration of field work): 1-4 months	• Impressions of university study and details can lose their clarity over time. On the other hand, the experience of study-ing can be reflected upon in a job-oriented context.	

Anhang 2 zu Giesler M, Huber J, Paulmann V. Quality assurance and curricular development of medical faculties using graduate surveys: Challanges - proposal of care questionnaire - implemention guide. GMS J Med Educ. 2022;39(1):Doc10. DOI: 10.3205/zma001531
